# Genome-wide association study and whole-genome sequencing identify a deletion in *LRIT3* associated with canine congenital stationary night blindness

**DOI:** 10.1038/s41598-019-50573-7

**Published:** 2019-10-02

**Authors:** Rueben G. Das, Doreen Becker, Vidhya Jagannathan, Orly Goldstein, Evelyn Santana, Kendall Carlin, Raghavi Sudharsan, Tosso Leeb, Yuji Nishizawa, Mineo Kondo, Gustavo D. Aguirre, Keiko Miyadera

**Affiliations:** 1 Department of Clinical Sciences and Advanced Medicine, School of Veterinary Medicine, University of Pennsylvania, Pennsylvania, United States of America; 2 Institute of Genome Biology, Leibniz Institute for Farm Animal Biology, Dummerstorf, Germany; 3 Institute of Genetics, University of Bern, Bern, Switzerland; 4 Baker Institute for Animal Health, College of Veterinary Medicine, Cornell University, Ithaca, New York, United States of America; 5 Department of Biomedical Sciences, Chubu University, Kasugai, Aichi Japan; 6 Department of Ophthalmology, Mie University Graduate School of Medicine, Tsu, Mie Japan

**Keywords:** Genome-wide association studies, Retina

## Abstract

Congenital stationary night blindness (CSNB), in the complete form, is caused by dysfunctions in ON-bipolar cells (ON-BCs) which are secondary neurons of the retina. We describe the first disease causative variant associated with CSNB in the dog. A genome-wide association study using 12 cases and 11 controls from a research colony determined a 4.6 Mb locus on canine chromosome 32. Subsequent whole-genome sequencing identified a 1 bp deletion in *LRIT3* segregating with CSNB. The canine mutant *LRIT3* gives rise to a truncated protein with unaltered subcellular expression *in vitro*. Genetic variants in *LRIT3* have been associated with CSNB in patients although there is limited evidence regarding its apparently critical function in the mGluR6 pathway in ON-BCs. We determine that in the canine CSNB retina, the mutant LRIT3 is correctly localized to the region correlating with the ON-BC dendritic tips, albeit with reduced immunolabelling. The *LRIT3*-CSNB canine model has direct translational potential enabling studies to help understand the CSNB pathogenesis as well as to develop new therapies targeting the secondary neurons of the retina.

## Introduction

Our ability to visually perceive the surrounding environment is a result of the concerted action of multiple specialized cell types in the retina. Photoreceptors are the photosensitive cells in the retina, consisting of rods responsible for vision in dim light, and cones that aid in colour perception and vision under bright light. Once the photoreceptors capture light energy, the information is converted into neural signals that are relayed to multiple types of bipolar cells (BCs) that in turn transmit the signal to tertiary retinal neurons such as amacrine and retinal ganglion cells, before being transmitted to the visual cortex. Based on their ability to either initiate or terminate light stimuli BCs can be either ON- or OFF-type. While cone photoreceptors can connect both ON- and OFF-BCs, the rods are served largely by the ON-BCs^1,2^.

Congenital stationary night blindness (CSNB) is a clinically and genetically heterogeneous, non-progressive disease that can occur due to genetic defects in retinoid metabolism in the retinal pigment epithelium (RPE), defects in photoreceptor phototransduction, or signal transmission through the ON-BCs^3^. Affected individuals exhibit non-progressive dark or dim-light visual difficulties (nyctalopia) starting from birth. However, these symptoms might be missed more often in the well-lit urban environment. Currently there is no cure for the disease. Based on the electroretinogram (ERG) patterns, CSNB can be clinically sub-classified as Riggs and Schubert-Bornschein forms. The a-wave and overall ERG amplitude are reduced, but the waveform is preserved, in the Riggs form due to defects in the photoreceptors^4^, and is linked to variants in genes underlying proteins involved in rod phototransduction. It has both autosomal dominant (*RHO*, *PDE6B* and *GNAT1*) and recessive (*SLC24A1* and *GNAT1*) inheritance^5–10^. The Schubert-Bornschein type has a “negative-type” ERG with a normal amplitude a-wave and a significantly reduced b-wave due to defective signalling from photoreceptors to the BCs^11^. In the incomplete form (incomplete CSNB), the signal transmission from photoreceptors to BCs is partially blocked and affects both the ON- and OFF-pathways, so patients have reduced but recordable residual rod function^12^. Genetic variants in *CACNA1F*^13,14^, *CABP4*^15^ and *CACNA2D4*^16^, which are all expressed in the photoreceptor synaptic terminals, can lead to incomplete CSNB. Finally, a dramatically reduced b-wave in the Schubert-Bornschein type is due to defects in the ON-BC pathway, resulting in complete CSNB. The mGluR6 receptor on ON-BCs detects decreased levels of glutamate released by photoreceptors in response to light, and the consequent deactivation of a G protein signalling cascade results in the closure of the TRPM1 cation channel, thereby evoking depolarization (activation) of ON-BCs. The loss of ERG b-wave in complete CSNB corresponds to the loss of this ON-BC depolarization. Genetic variants that can cause complete CSNB have been found in *NYX*^17,18^, *GRM6*^19^, *TRPM1*^20,21^, *GPR179*^22,23^, and *LRIT3*^24^, whose protein products all localize to the dendritic tips of the ON-BCs.

Model organisms are important for both basic and translational research, as they provide critical clues not only for a better understanding of the disease and its mechanism, but are also used to develop new therapies. A series of transgenic mouse models of complete CSNB (also known as the no b-wave (nob) mice) have been developed including *Nyx*^nob1^ ^25^, *Cacna1f*^nob2^ ^26^, *Grm6*^TmlNak^ ^27^, *Grm6*^nob3^ ^28^, *Grm6*^nob4^ ^29^, *Gpr179*^nob5^ ^30^, and *Lrit3*^nob6^ ^31^. Further, spontaneously-occurring mouse models such as *Grm6*^nob7^ ^32^, *Grm6*^nob8^ ^33^ and *Lrit3*^tvrm257^ ^34^ have also been described. Naturally-occurring complete CSNB has also been reported in Appaloosa horses and at least two other equine breeds with a white spotting phenotype (leopard complex spotting)^35^ which have a homozygous 1378-bp retroviral insertion in intron 1 of *TRPM1*^36^.

We have previously reported the first naturally-occurring canine model of complete CSNB with an autosomal recessive mode of inheritance segregating in a research colony of Beagle dogs^37^. Herein we report a genome-wide association study (GWAS) which, together with whole-genome sequencing (WGS) performed in parallel, identified an association between canine CSNB and a truncating single base deletion in *LRIT3*, a gene that has previously been associated with CSNB in mice and people. Of the 30+ canine inherited retinal disease models whose causative genetic variants have been identified to date, the clinically-relevant canine *LRIT3*-CSNB is the only model with ON-BC defects and therefore carries unique translational potential in therapeutic targeting of these secondary retinal neurons.

## Results

### GWAS mapping of a 4.6 Mb locus on CFA32 associated with canine CSNB

**Figure 1:**
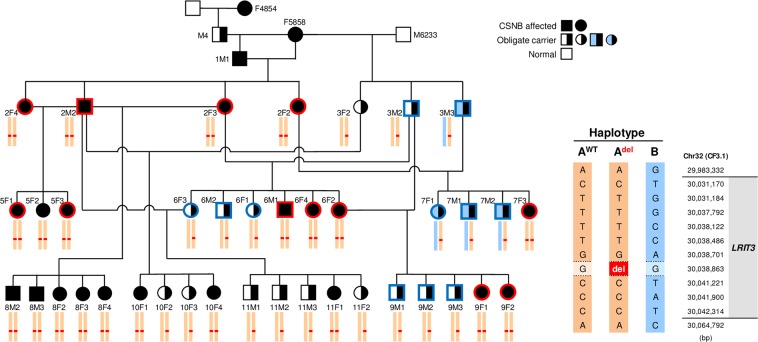
Segregation of CSNB in the canine research colony and association with the haplotype harboring the *LRIT3* variant. The pedigree of the canine research colony segregating the autosomal recessive CSNB phenotype. The symbols for the animals included in the GWAS are outlined in bold red (cases) and blue (control carriers). The diploid haplotype spanning the canine *LRIT3* genomic region is shown under each symbol or animal ID in all animals whose DNA sample was available. The details of the three existing haplotypes defined by informative SNV markers are shown in the right side of the figure along with their chromosomal locations. Two haplotypes A^WT^ (orange block) and B (light blue block) were identified initially. Subsequent fine mapping of the region using whole-genome sequencing identified additional sequence variants including a 1 bp deletion highlighted in red (chr32:30,038,863; CanFam3.1), defining a third haplotype A^del^. The 1 bp deletion in *LRIT3* segregated completely with the CSNB phenotype.

**Figure 2:**
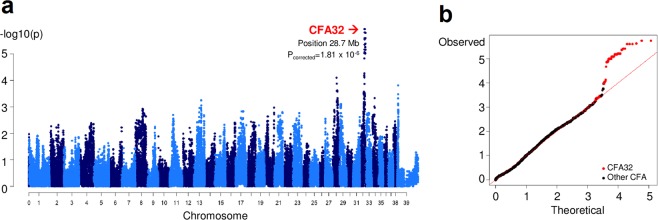
Mapping of canine CSNB by genome‐wide association study. (**a**) Genome-wide association plot using 12 CSNB cases, and 11 obligate carriers as controls. Note the association signal on canine chromosome 32 (CFA32). (**b**) The quantile–quantile (QQ) plot shows the observed *vs* expected -log *P* values. The straight line in the QQ plot indicates the distribution of SNV markers under the null hypothesis, and the skew at the right edge indicates those markers that are more strongly associated with the trait than would be expected by chance. Markers from CFA32 are shown in red.

**Figure 3:**
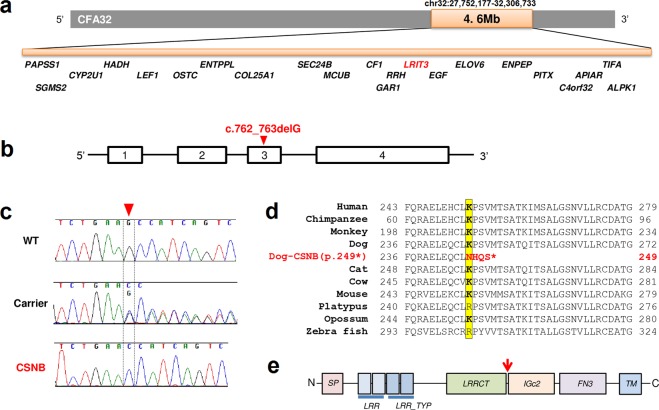
The mapped CSNB interval and the genetic variant in *LRIT3* leading to a premature stop codon. (**a**) The 4.6 Mb mapped CSNB interval (shaded in orange) in canine chromosome 32 (CFA32) shown with the 22 genes localized in the region. (**b**) The 1 bp deletion is located in exon 3 of *LRIT3*. (**c**) Representative chromatograms showing the c.762_763delG variant in heterozygous and homozygous states in the carrier and CSNB, respectively, while it is absent in WT. (**d**) Alignment of LRIT3 across different species indicating high evolutionary conservation of the immunoglobulin c2 type (IGc2) domain where the 1 bp deletion was identified (highlighted in yellow). The sequences used for alignment are: XP_005156597.2 (Zebra fish), XP_007668336.1 (Platypus), XP_007496058.1 (Opossum), AHI87499.1 (Mouse), NP_001178443.1 (Cow), XP_005555723.1 (Monkey), AAI04038.1 (Human), XP_001139031.3 (Chimp), XP_853150.3 (Dog) and XP_003985132.1 (Cat). (**e**) Domains of canine LRIT3 as predicted by Scan Prosite (https://prosite.expasy.org). The red arrow indicates the location of the deletion. SP, signal peptide; LRR, leucine rich repeat; LRR_TYP, LRR type; LRRCT, LRR C-terminal; IGc2, immunoglobulin c2 type; FN3, fibronctin type 3; TM, transmembrane.

GWAS was performed using DNA samples from 12 CSNB and 11 control animals from a canine research colony (Fig. 1). Note that all the controls available were obligate carriers as a result of an intentional breeding strategy aimed at producing CSNB affected dogs. After pruning the single-nucleotide variant (SNV) chip data for low genotyping rate and low minor allele frequency, we identified a single strong hit on canine chromosome 32 (CFA32) with a corrected p-value of 1.81 × 10^−6^ (Fig. 2a). The quantile-quantile plot of observed versus expected p-values of the mixed model analysis also supported the effectiveness of the correction for population structure and the significance of the CFA32 locus (Fig. 2b). Of the 2,908 SNVs on the genotyping chip designed to cover CFA32, there were 2,135 SNVs that proved to be informative using the above sample population. The top 10 SNVs with the highest association achieving a corrected p-value of ≤5.75 × 10^−5^ resided on CFA32 between 22,654,843 and 28,799,548 bp. Since the CSNB allele was introduced in the research colony by a common founder, we hypothesized that the affected animals would be identical by descent (IBD) for the causative genetic variant as well as for the flanking chromosomal segments. Therefore, homozygosity mapping was performed on CSNB cases to search for extended regions of homozygosity with simultaneous allele sharing on CFA32. Consequently, we identified a single chromosomal region of homozygosity spanning 4.6 Mb (CFA32: 27,752,177-32,306,733) (Fig. 3a; Supplementary Fig. 1).

### Identification of a 1 bp deletion in *LRIT3* by WGS

To comprehensively screen for variants across the critical interval, as well as for surveying the entire genome for chromosomal segments and pathogenic genetic variants that could not be identified by the SNV-chip based approach, WGS was carried out. Paired-end reads (2 × 100 bp) were collected from a shotgun DNA fragment library from two CSNB affected and two carrier animals (European Nucleotide Archive accession # ERS3011607-#ERS3011610), achieving genome-wide coverages of 13 × −20x for each sample. Single nucleotide and indel variants were called against the canine reference genome (CanFam3.1). The variants in the two CSNB cases were filtered against the genomes of the two carriers as well as 271 dogs from 75 different breeds in which CSNB was not known to segregate, and had been sequenced for unrelated studies (Supplementary Table 1). We hypothesized that the causative variant should be homozygous in the CSNB cases, heterozygous in the carriers, and absent in the control dogs of other breeds. After a genome-wide filtering of variants, a single variant with the strongest predicted pathogenic effect fitting these characteristics was identified; a 1 bp homozygous deletion (CFA32: 30,038,862_30,038,863delG) in *LRIT3* (Fig. 3b,c; Supplementary Fig. 2). This variant was expected to cause a frameshift with early protein truncation (p.249*) (Fig. 3d) in both LRIT3 isoforms (681 aa. and 493 aa.) (Supplementary Information 1) expressed in the retina. Genotyping of DNA samples of all the research dogs available from the CSNB pedigree confirmed that this deletion was in complete concordance with the CSNB disease status clinically assessed by ERG (Fig. 1). Further, we have confirmed complete co-segregation of the *LRIT3* variant with CSNB in a more recently developed satellite research colony of 39 additional dogs (Supplementary Fig. 3) originating from 3 of the affected dogs in the original research colony used for GWAS.

In a previous study, we excluded *LRIT3* from causal association with CSNB through haplotype analysis based on 13 informative genetic markers (11 intragenic and 2 flanking) identified across the genomic region of *LRIT3*^37^. However, this approach failed to identify the disease association of *LRIT3* due to its inability to differentiate the disease chromosome (A^del^) from one of the non-disease chromosomes (A^WT^). This is likely due to **a**-limited number of markers used, and **b**-minimal difference in marker pattern between chromosomes A^del^ and A^WT^ due to the relatively recent emergence of the disease variant. Further, in the same study, we had described the exclusion of exonic sequence variants in *LRIT3* based on Sanger sequencing^37^. Upon careful re-examination of experimental records, we determined a critical misidentification of samples interfering with the correct interpretation of the sequencing results. Once the appropriate sample was assigned, the *LRIT3* homozygous deletion (CFA32: 30,038,862_30,038,863delG) identified by WGS in the current study was indeed confirmed to segregate completely with CSNB.

The canine LRIT3 is predicted to have a signal peptide, two LRR (Leucine-rich repeats), two LRR type domains, and one each of LRRCT (LRR C-terminal), IGc2, FN3, and transmembrane domains. The truncated protein is predicted to lack the IGc2, FN3, and the C-terminal transmembrane domains (Fig. 3e). Comparative amino acid sequence alignment of LRIT3 across species showed that the four altered amino acids before the premature stop codon in the canine mutant are well conserved across the different species examined, with only minor variations found in platypus and zebrafish (Fig. 3d).

### RNA-seq data reveal the expression of the mutant *LRIT3* transcript and unremarkable expression profile of genes in the mGluR6 pathway

In mammalian cells, transcripts containing premature stop codons are generally degraded by nonsense-mediated decay (NMD). The efficiency, however, depends on the position of the premature stop codon^38^. To examine if the genetic variant in *LRIT3* promotes NMD, we utilized RNA-seq data obtained from CSNB, carrier and WT retinal samples (n = 3 each). These 75 bp single-end RNA-seq data were also utilized to assess differential expression of genes across the retinal transcriptome. When individual bam files were visualized using the Integrative Genomics Viewer (IGV), the alignments represented full-length *LRIT3* transcripts in WT, carrier, and CSNB samples (Supplementary Fig. 2A), indicating that the mutant transcript with the premature stop codon escapes NMD. Transcriptome-wide analysis did not reveal any retinal expressed genes to be differentially expressed between WT, carrier, and CSNB. This included *LRIT3* and other genes in the mGluR6 pathway (Supplementary Fig. 4A). At the protein level, TRPM1, the critical channel protein of the mGluR6 pathway and localized by LRIT3, was expressed similarly in WT, carrier, and CSNB (Supplementary Fig. 4B). These findings suggest that the disease was impacted specifically by the loss of function of the mutated LRIT3.

### Experimental identification of the canine *LRIT3* transcript sequence and its isoform

**Figure 4:**
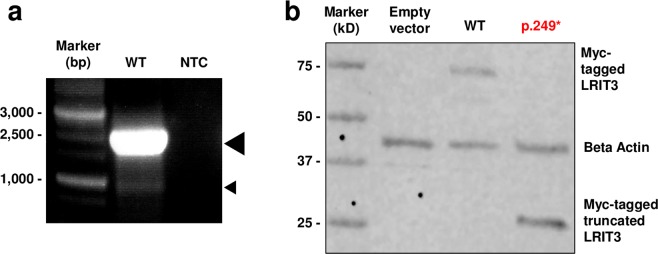
WT canine *LRIT3* transcripts and expression of WT and mutant canine LRIT3 proteins. (**a**) RT-PCR showing the presence of full-length *LRIT3* (large arrow head) as well as a shorter and significantly less abundant second isoform (small arrow head) in the WT canine retina. NTC, no template control. (**b**) Western blot showing expression of the expected full product (75 kD) with WT LRIT3 while the mutant LRIT3^p.249*^ show expression of a truncated protein (29 kD) and lack of the full product. Images of the complete gel (**a**) and blot (**b**) are included in Supplementary Fig. 7.

The canine *LRIT3* cDNA sequence was derived from our canine retinal RNA-seq data and was confirmed to consist of four exons. This differed from the NCBI prediction that lacked the first, non-coding exon, as well as from the Ensembl prediction (ENSCAFT00000018278.3; accessed 11/21/2018) that lacked a larger portion of the 5′ exon, thereby predicting truncation of the 5′ end of the transcript. The experimentally confirmed canine *LRIT3* cDNA sequence is shown in Supplementary Information 1. Based on the revised canine *LRIT3* transcript sequence, primers were designed to clone the full coding sequence of canine *LRIT3*. RT-PCR yielded the full-length transcript (2,080 bp) as well as a smaller, less abundant product (1,607 bp) (Fig. 4a). Both products were gel-purified and Sanger sequenced. Alignment of the two sequences revealed a splicing event in which exon 2, consisting of 473 bp, had been spliced out in the shorter product, confirming the presence of two *LRIT3* isoforms in canine retina. As the larger, full-length transcript was predominant among the transcripts and contained all the elements, subsequent functional studies were carried out using this 2,080 bp transcript.

### Comparable subcellular localization between WT and mutant canine LRIT3

To examine the subcellular localization of the expressed LRIT3 protein and determine if the mutant protein was trapped in the endoplasmic reticulum (ER) or Golgi, WT and mutant canine *LRIT3* cDNA were each cloned into expression vectors with N-terminal Myc tags, and overexpressed in mammalian cultured cells, COS1 and 661 W. Transfected cells were either harvested for protein extraction and used for Western blot or fixed with 20% paraformaldehyde for immunocytochemistry. Immunoblotting with anti-Myc showed the expected 75 kD band in the WT while a truncated fragment just above the size of 25 kD was found for the mutant LRIT3 (predicted to lack the IGc2, FN3, and C-terminal transmembrane domains and having a molecular weight of 29 kD) (Fig. 4b). Therefore, we experimentally confirmed that the stop codon introduced by the deletion in *LRIT3* gave rise to a truncated protein while escaping NMD. Immunocytochemistry in COS1 cells did not find notable differences in subcellular localizations between WT and mutant LRIT3. Co-immunolabelling with cellular markers indicated no abnormal retention of the mutant protein in the ER or Golgi (Supplementary Fig. 5).

### Reduced punctate LRIT3 immunolabelling in the OPL of canine CSNB

**Figure 5:**
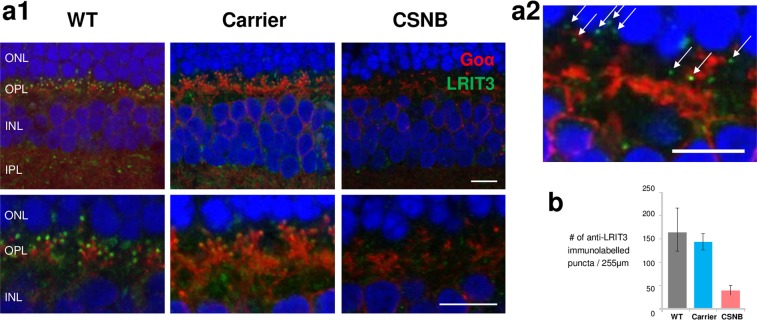
Decreased LRIT3 immunolabelling in the region correlating with the ON-BC dendritic tips in the *LRIT3*^del/del^ CSNB retina. (**a1**) Representative confocal microscopy images of canine retinal cryosections showing distinct LRIT3 puncta immunolabelling in the region correlating with the dendritic tips of ON-BCs (labelled with anti-Goα antibody) in WT and carrier retinas. In the CSNB canine retina, these signals are decreased in intensity and numbers (see B). The lower panels are magnified images of the ON-BC dendrites. Scale bar, 10 µm. (**a2**) High magnification of the OPL in the CSNB retina from (**a1**) to display the punctal labelling (arrows) in the region correlating with to the ON-BC dendritic tips. Scale bar, 10 µm. (**b**) The number of LRIT3-labelled puncta in the region correlating with the ON-BC dendritic tips was counted in retinal sections co-labelled with anti-LRIT3 and Goα antibodies. While the WT and carrier retinas showed comparable number of puncta, the number was markedly reduced in the CSNB retina (<1/3 of WT and carrier). Note that statistical analysis could not be performed due to small sample sizes. Error bars represent the range of counts.

We have previously carried out a detailed IHC to determine if retinal morphology and localization of relevant proteins are affected in CSNB^37^. In that study, our IHC conditions used did not allow effective immunolabelling of retinal cryosections with the anti-LRIT3 antibody. To this end, we have modified the antigen retrieval method for IHC and now can detect distinct punctate immunolabelling in the outer half of the OPL in the WT retina (Fig. 5a). When co-immunolabelled with Goα, these puncta were localized to the region correlating with the rod and cone ON-BC dendritic tips. While intense punctate immunolabelling was found in the WT retinas, the labelling was less intense in the carrier and markedly reduced in CSNB retinas (Fig. 5a). Further, while the number of puncta was comparable between the WT and carrier retinas, the CSNB retina had reduced (<1/3^rd^) punctate labelling (Fig. 5b), and had less stringent localization of specific LRIT3 labelling to the region correlating with the dendritic tips. These observations are expected given that the commercial anti-LRIT3 antibody has reduced epitope matching with the truncated mutant LRIT3 (55 amino acids) in contrast to the WT LRIT3 (141 amino acids). Notably, as we have shown previously, immunolabelling of PKCα is altered in the CSNB retina where there was reduced labelling intensity in the somata^37^ as well as in the dendrites (Supplementary Fig. 6). These findings are compatible with the functional defect of the mGluR6 pathway taking place in the ON-BC dendrites of CSNB retinas.

### Altered peanut agglutinin (PNA) labelling along the OPL of canine CSNB

**Figure 6:**
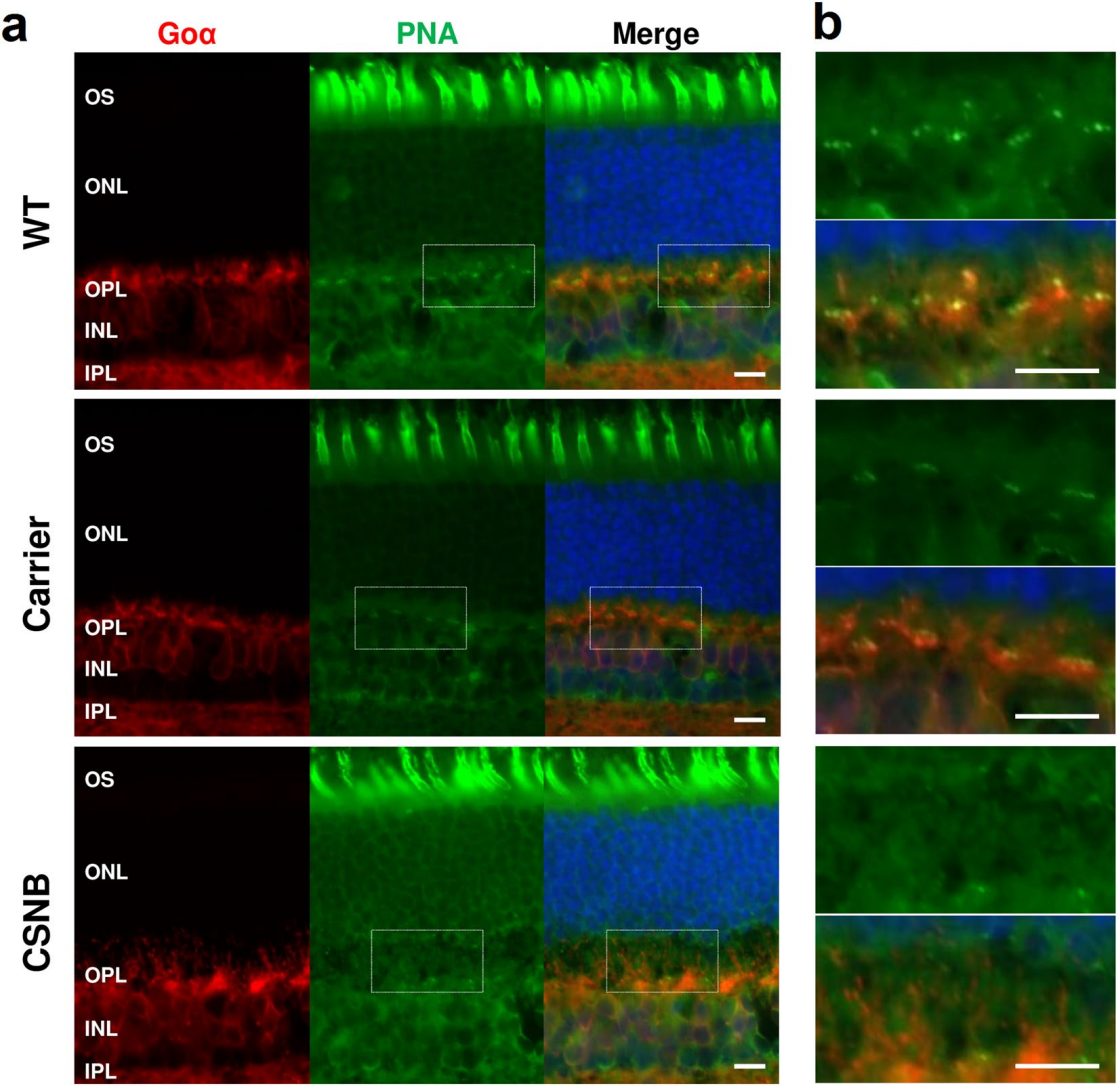
Altered PNA staining in the OPL of the CSNB retina. (**a**) Cryosections of WT, carrier and CSNB affected canine retinas co-labelled with anti-Goα antibody (red) and PNA (green). The organized PNA labelling present along the OPL was evident in WT and carrier retinas but not in the CSNB retina. There was no significant difference in the PNA labelling pattern associated with the cone outer segment across the three sample groups. (**b**) Magnified images of the OPL outlined in (a) are shown. Distinct staining with PNA is horizontally organized along the ON-BC dendritic tips in the WT retina. Similar pattern, albeit slightly less intense, is present in the carrier retina, but is minimal in the CSNB retina. Scale bar, 10 μm.

PNA is a marker that labels the insoluble extracellular matrix surrounding cone photoreceptor outer and inner segments as well as the synaptic cleft^39–41^. To determine if there are PNA labelling abnormalities at the OPL level^42^, we examined the PNA labelling pattern in WT, carrier and CSNB canine retinas, counterstained with the ON-BC marker Goα (Fig. 6a,b). In the WT canine retina we found organized clusters of intense PNA labelling aligned horizontally along the OPL, and overlapping with the ON-BC dendritic tips (Fig. 6b). In contrast, the CSNB retina had fewer, less intense, and disorganized PNA labelling along the OPL. The carrier retina seemed to retain the horizontal organization of the PNA labelling. These findings indicate that the architecture of the cones and the contacting ON-BCs is distorted and that the synaptic conformation is altered in the CSNB retina.

### Comparable ultrastructure of photoreceptor to ON-BC synapses between WT and CSNB canine retinas

**Figure 7:**
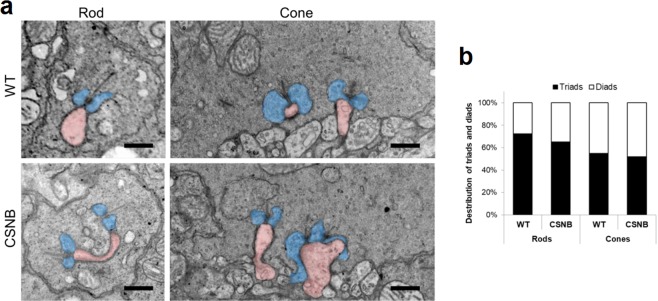
Comparable ultrastructure of rod and cone synapses in WT and CSNB canine retina. (**a**) Ultrastructure of rod and cone synapses in WT and CSNB canine retinas. Representative transmission electron microscopy images are shown for rod and cone ribbon synapses. Invaginating dendrites (pink) of both rod and cone ON-BCs, and horizontal cell processes (blue) are illustrated. Scale bar, 500 nm. (**b**) Quantification of synaptic elements identified in rod spherules and cone pedicles for each WT and CSNB canine retina. Data is shown as the ratio (%) of diads and triads for each rod and cone synapses in which the number of diads and triads were counted. Synapses were classified as diads if they have identifiable ribbon with adjacent horizontal cell process (blue). If there is additional ON-BC process (pink) next to the synaptic ribbon, they were designated as triads.

To examine if the synaptic contacts of invaginating ON-BC dendrites were altered^43^, we examined the ultrastructure of WT and CSNB canine retinas by transmission electron microscopy. We found that both cone pedicles and rod spherules were formed and localized normally in the affected canine retina, consistent with the observation by others in the *Lrit3*^nob6/nob6^ mouse^43^. We also found that the invaginating ON-BCs formed triads across the cone and rod populations with no significant difference between WT and mutant canine retinas (Fig. 7a). This is in contrast to the *Lrit3*^nob6/nob6^ mouse that was found to have decreased triads in the cone pedicles^43^. In addition, we found no notable differences with the OFF-BC contacts between WT and mutant canine retinas. To quantitate the contact elements, we identified 76 and 83 rods spherules, and 23 and 46 cone pedicles in non-overlapping sections from WT and CSNB canine retinas, respectively, and demonstrated that the distribution of triads and diads was comparable between WT and CSNB canine retinas for both rods (p = 0.68) and cones (p = 0.90) (Fig. 7b).

## Discussion

We have previously characterized an autosomal recessive form of complete CSNB in a research colony of Beagle dogs, and excluded the functional candidate genes from disease association^37^. In the present study, we carried out a GWAS to map the disease locus, followed by WGS which led to the identification of a single base deletion (c.762_763delG) in *LRIT3*, one of the genes previously excluded. That exclusion was based on haplotype analysis using 13 informative genetic markers (11 intragenic and 2 flanking) across *LRIT3*, as well as Sanger sequencing of exons^37^. We have now confirmed that the haplotype analysis was unable to detect the disease association with *LRIT3* due to insufficient marker density. Further, we have determined that there was a critical sample misidentification in the previous study leading to the *LRIT3* variant (CFA32: 30,038,862_30,038,863delG) being disregarded from association. Typing of the *LRIT3* variant in all available dogs in the CSNB research colony as well as in a more recently developed satellite colony confirmed complete co-segregation of the *LRIT3* variant with the CSNB phenotype. Furthermore, the variant was not present in any of the 271 control dogs representing 75 breeds derived from the Dog Biomedical Variant Database Consortium.

The CSNB-associated variant identified in *LRIT3* introduces a premature stop codon in LRIT3 (p.249*). RNA-seq shows the presence of full-length *LRIT3* mRNA in the mutant retina, suggesting escape from NMD. Cloning and *in vitro* expression of the mutant LRIT3 demonstrated the presence of a truncated protein of approximately 29 kD. *In vitro* cellular expression pattern was comparable between WT and mutant LRIT3 with no evidence of entrapment of mutant protein in the ER or Golgi. We would note that while the *in vitro* studies provide an opportunity to test such hypotheses, overexpression in undifferentiated cultured cells that are not ON-BC can cause departure from the natural expression patterns, and hence caution is required upon interpretation of the data.

At the retinal level, IHC showed specific localization of anti-LRIT3 immunolabelling in the region correlating with the ON-BC dendritic tips of the controls retinas, while the LRIT3 signal was reduced in intensity and number in the CSNB retinas. It is important to note that the commercial anti-LRIT3 antibody utilized recognized a 141 amino-acid epitope that spanned the site of the 1 bp deletion and protein truncation. As a result, only the N-terminus 37% (52/141 amino acids) of the epitope was present in the mutant LRIT3 followed by 4 aberrant amino acids before the stop codon. Further to the partial loss of epitopes recognized by the antibody, the smaller size of the truncated mutant LRIT3 could be contributing to the reduced LRIT3 signal in IHC. Finally, the altered immunolabelling of rod ON-BC dendrites with anti-PKCα supports the basis for ON-BC dysfunction observed clinically as well as electroretinographically. As the mutant LRIT3 lacks the IgC2, serine-rich FN3 and transmembrane domains, further investigation of the functional consequences of these deficits at the molecular level will shed light on the poorly understood role and interacting partners of LRIT3 within the mGluR6 pathway. For example, LRIT3 is thought to play a supportive role in the ability of nyctalopin to localize TRPM1 to the dendritic tips of BCs^42,44^. In support, the *Lrit3*-deficient nob6 mice lack most of the post-synaptic clustering of mGluR6 pathway components, although this abnormality is limited to cone ON-BCs^42^. More recent TEM data suggest that LRIT3 might be involved in the coordination of the trans-synaptic communication between cones and ON-BCs during synapse formation and function^43^. In the *LRIT3*-CSNB canine retina, we found that mGluR6 pathway components were still present, although they were less abundant compared to the WT^37^. In the current study, we find attenuated PNA labelling along the OPL in canine CSNB as was found in the *Lrit3* mutant mouse^43^. However, contrary to findings in the *Lrit3* mutant mouse, ultrastructural abnormalities specific to the cone synapses are not found in the canine CSNB retina indicating differences in pathogenesis presumably affected by the type of *LRIT3* disease variant (i.e. partial C-terminus truncation in the canine mutant or complete deficiency in the knockout murine model).

While this manuscript was under review, Hasan *et al*. published a study in the mouse retina proposing a transsynaptic role of LRIT3^45^. They demonstrated by *in situ* hybridization that LRIT3 is expressed rather presynaptically in the rod photoreceptor terminals and acts transsynaptically to localize the TRPM1 channel in the postsynaptic bipolar cells. Further, they showed ERG recovery in knockout mice when LRIT3 expression was restored in the rods via AAV delivery. The resolution of IHC used, however, was insufficient to allow differentiation between pre- and post-synaptic localization of LRIT3 which appeared to ‘colocalize’ with mGluR6 and TRPM1 in the mouse retina. Studies are underway to verify LRIT3 localization in the canine retina to identify the therapeutic target cells for reversing the CSNB phenotype. In summary, we have identified a single base deletion in *LRIT3* associated with complete CSNB in the canine model. The mutant *LRIT3* transcript escapes NMD and gives rise to a truncated protein that shows unaltered subcellular expression in cultured cells *in vitro*. In the canine CSNB retina, immunolabelling of LRIT3 correlating to the ON-BC dendritic tips is altered with reduction in intensity and quantity, and there is altered immunolabelling of rod ON-BC dendrites. Future studies aim to elucidate the molecular pathogenesis as well as to test therapeutic targeting to restore ON-BC function.

## Materials and Methods

### Ethics statement

The research was conducted in full compliance and strict accordance with the Association for Research in Vision and Ophthalmology (ARVO) Resolution on the Use of Animals in Ophthalmic and Vision Research. The protocol was approved by the Institutional Animal Care and Use Committees (IACUCs) of Mie University Graduate School of Medicine (#24–49) and the University of Pennsylvania (#801870). Even though none of the procedures used were expected to cause pain to the study subjects, every effort was made to eliminate potential discomfort.

### Animals, genome-wide association study, and homozygosity mapping

Genomic DNA samples from a subset of Beagle dogs from a canine research colony described previously^37^ – 12 CSNB cases (10 females, 2 males) and 11 obligate carriers (3 females, 8 males) were genotyped on the Illumina CanineHD BeadChip (Illumina, San Diego, CA) containing 173,662 SNV markers. The data were subjected to standard quality control, where SNVs were excluded for poor genotyping rates (<95%) and low minor allele frequencies (<0.01). Population stratification, resulting from close familial relationships, was confirmed by the genomic inflation factor of 1.55 calculated during an association test. Therefore, a mixed model approach utilizing the GenABEL package^46^ was applied for the association analysis to correct for this population stratification and any cryptic relatedness. This correction resulted in a genomic inflation factor of 1.04. To identify extended homozygous regions with allele sharing across all affected animals using the software PLINK v1.07^47^ (http://pngu.mgh.harvard.edu/purcell/plink/), the options–homozyg-group and–homozyg-match were applied. Haplotypes around the significantly associated locus were constructed using PHASE^48^. The final critical interval was defined by visual inspection of all SNV chip genotypes of the cases on canine chromosome 32 in an Excel-file. All genome positions refer to the CanFam3.1 reference sequence assembly.

In addition to the dogs from the original colony^37^, 39 dogs from a newly developed sattellite colony were included for the genotype-phenotype segregation analysis. The founder of this satellite colony included 3 animals from the original colony (Supplementary Fig. 3). Additional founders included Beagle dogs obtained from a commercial laboratory animal vendor and that were identified as carriers or affected for CSNB.

### Whole-genome sequencing and variant calling

Genomic DNA fragment library with a 250 bp insert size was prepared from two CSNB affected (1 female, 1 male) and two obligate carriers (1 female, 1 male) using an Illumina TruSeq PCR-free DNA Library Preparation Kit. Whole-genome sequencing was carried out using the HiSeq. 2500 instrument (Illumina, San Diego, CA). The number of paired-end reads (2 × 100 bp) for each of the four samples were 120, 172, 230, and 239 million reads corresponding to a mean mapped read coverage of 14.59, 20.82, 27.89 and 28.96 times depth, respectively. The reads were mapped to the dog reference genome (CanFam3.1) using the Burrows-Wheeler Aligner (BWA) version 0.7.5a^49^ with default settings. The Picard tools (http://sourceforge.net/projects/picard/) were used to sort the mapped reads by the sequence coordinates and to label the PCR duplicates. The Genome Analysis Tool Kit (GATK version v3.6)^50^ was used to perform local indel realignment followed by base quality recalibration (BQSR). Finally, single-nucleotide variants (SNVs) and indels were called using the GATK 2-step process using ‘HaplotypeCaller’ and ‘GenotypeGVCFs’. GenotypeGVCFs was run per-chromosome (combined afterward using GATK ‘CatVariants’). Variant data was obtained in variant call format (version 4.0) as raw calls for all samples and sites flagged using the variant filtration module of GATK. Variant calls that failed to pass the following filters were labelled accordingly in the call set: (i) Hard to Validate MQ0 ≥ 4 & ((MQ0/(1.0 * DP)) > 0.1); (ii) strand bias (low Quality scores) QUAL < 30.0||(Quality by depth) QD < 5.0||(homopolymer runs) HRun > 5||(strand bias) SB > 0.00; (iii) SNP cluster window size 10. The snpEFF software^51^ together with the CanFam 3.1 annotation was used to predict the functional effects of detected variants. We considered the following snpEFF categories of variants as non-synonymous: non_synonymous_coding, codon_deletion, codon_insertion, codon_change_plus_codon_deletion, codon_change_plus_codon_insertion, frame_shift, exon_deleted, start_gained, start_lost, stop_gained, stop_lost, splice_site_acceptor, splice_site_donor. The critical interval on chromosome 32 contained 4,554,556 bases with a depth average of 21.6. The sequence data from the CSNB cases and carriers were compared to the Boxer reference genome (CanFam3.1) and whole-genome sequencing data from 271 dogs not known to be affected with CSNB representing 75 breeds in the Dog Biomedical Variant Database Consortium (University of Bern, Zurich) (Supplementary Table 1).

### Tissue collection, RNA and protein isolation

Whole retinas were isolated from the posterior eyecups of three CSNB affected (females; age 13-25 mon), three carrier (females, age; 13–25 mon), and three WT (1 male, 2 females; age 7–14 mon) animals following immediate collection of the globes after humane euthanasia, flash frozen in liquid nitrogen, and stored at −80 °C. RNA was extracted from retinal tissues using conventional Trizol method followed by DNase I treatment (Ambion, Carlsbad, CA). Total protein was extracted from the retina of the fellow eyes of the same group of CSNB and carrier animals as well as 3 other WT controls (1 male, 2 females; age 7–25 mon) using T-PER™ reagent (Thermo Fisher Scientific, Waltham, MA) with 1% protease and phosphatase inhibitor cocktail (Thermo Fisher Scientific, Waltham, MA).

### Canine retinal library preparation, RNA-seq data generation, sequence alignment, abundance estimation, data normalization, and data visualization

Retinal samples of three each CSNB, carrier, and WT control animals described under tissue collection were used for RNA-seq. Prior to cDNA library preparation, RNA integrity number (RIN) was assessed using 2100 bioanalyzer (Agilent, Santa Clara, CA) to determine the quality of the RNA. Poly(A) + -enriched cDNA libraries were generated using the TruSeq Sample Preparation kit (Illumina) and checked for quality and quantity using the bioanalyzer and qPCR (KAPA Biosystems, Wilmington, MA). Single-end reads (76 bp) were obtained using the Illumina HiSeq. 1500 platform. Trimmomatic^52^ was used to remove any remaining Illumina adapter sequences from reads and to trim bases off the start or the end of a read when the quality score fell below a threshold of 20. Sequence quality metrics were assessed using FastQC (www.bioinformatics.babraham.ac.uk/projects/fastqc/). Sequence reads were aligned to the canine reference genome (CanFam3.1) using STAR^53^. The abundance of reads mapping to each gene feature in the aligned genome was determined using HTSeq^54^. Non-expressed and weakly expressed genes, defined as having less than 1 read per million in the three samples per group were removed for further analysis. A correlation plot was generated to determine the correlation between technical replicates. The technical replicates of each sample were then merged into one file. This was followed by counting the number of genes and quality assessment using RseqC^55^. Sequences aligned with the canine reference genome were visualized using the IGV program.

### Global data assessment and differential expression analysis of RNA-seq data

Quantile normalization was applied to all samples^56^ and data were log2-transformed. To evaluate replicates and visualize the relationships between samples, Pearson correlation and Principal Component Analysis (PCA) were used. Multiple approaches were used to identify differentially expressed genes (DEGs) between different groups, including DESeq2^57^, edgeR^58^ with or without removing unwanted variations (RUV)^59^. Genes with a Benjamini-Hochberg (BH) multiple testing adjusted P value of <0.05 were defined as differentially expressed.

### Western blot analysis

Western blots were carried out as previously described^60^. Details of the primary antibodies (anti-TRPM1, -Myc1, and -ACTB antibodies) are shown in Supplementary Table 2. Despite multiple attempts with varying conditions, Western blot using the anti-LRIT3 antibody was unsuccessful resulting in non-specific reactions with the canine samples. We therefore chose Myc-tags for the identification of LRIT3 products *in vitro*. Briefly, retinal or cell lysates were quantitated using the BCA Protein Assay Kit (Thermo Fisher Scientific, Waltham, MA). Experiments were done in triplicate with 30 μg of protein loaded per lane. The immunoblot was scanned on the Odyssey Fc Dual-Mode Imaging System (LI-COR, Lincoln, NE) and normalized against β-actin using the LI-COR Image Studio Software (LI-COR). For each sample, the average of the three β-actin-normalized values per each antibody was compared with that of the corresponding normalized and averaged value of the WT sample and expressed as fold-changes. Statistical significance was calculated by an unpaired t-test using GraphPad (GraphPad Software, San Diego, CA).

### Retinal histology, immunohistochemistry (IHC), and electron microscopy

Retinal cryosections from CSNB affected (two females; 13 and 25 mon), carrier (two females; 13 and 25 mon), and three age-matched WT control animals were analyzed as described previously^37^. The antibodies used in this study are listed in Supplementary Table 2. Note that the epitope sequence for the commercial anti-LRIT3 antibody used (#HPA013454, Sigma-Aldrich, St. Louis, MO) spanned the mutation site. This resulted in only the first 52 amino acids of the epitope being present in the mutant LRIT3 whereas the full 141 amino acid epitope sequence was present in the WT canine LRIT3. Immunolabelled slides were examined with a Nikon A1R confocal microscope (Nikon Instruments Inc., Melville, NY) at 60x and 100x, and digital images were captured and processed using the NIS Elements software (Nikon Instruments Inc., Melville, NY). The number of anti-LRIT3 labelled puncta in the outer plexiform layer (OPL) in an area equidistant from the optic nerve edge and the ora ciliaris retinae were counted in the respective samples following image capture at 60x resolution in a confocal microscope. The mean of the total numbers of LRIT3 puncta was calculated and compared among the different *LRIT3* genotypes. Ultrastructure of the cone pedicles and BCs were imaged by transmission electron microscopy (TEM) with a JEM 1010 electron microscope (JEOL USA, Inc., Peabody, MA) fitted with a Hamamatsu digital camera (Hamamatsu Photonics, Bridgewater, NJ) and AMT Advantage image capture software (Advanced Microscopy Techniques, Woburn, MA). The epoxy resin-embedded samples for TEM had been prepared for the previous study^37^. Statistical analysis of the distribution of the contact elements (i.e. triads and diads) for both rods and cones between WT and CSNB retinas was done by two-tailed Chi-square test with Yates correction using GraphPad (GraphPad Software, San Diego, CA).

### DNA constructs, cell transfection, protein analysis and localization of LRIT3

Retinal RNA was extracted from CSNB-affected and WT canine retinas and cDNA was generated by reverse transcription using SuperScript IV VILO Master Mix (Thermo Fisher Scientific, Waltham, MA). Full-length canine *LRIT3* was cloned into the pKMyc vector (Addgene, Cambridge, MA)^61^ for each cDNA sample to create N-terminal Myc-tagged mutant and WT *LRIT3* constructs. Since the transcript cloned from a WT canine retina had two SNVs that differed from the CanFam3.1 reference genome at codons 561 and 1271 that altered the amino acids which were relatively well conserved in the reference genome, QuikChange II XL Site-Directed Mutagenesis kit (Agilent, Santa Clara, CA) was used to introduce the base changes at the two positions. African green monkey kidney fibroblast-like cell line (COS1; ATCC, Manassas, VA; cat. #CRL-1650; lot #59102713) and mouse cone photoreceptor-like cell line (661 W; purchased from Dr. Muayyad Al-Ubaidi, University of Houston) were grown to 80% confluency in DMEM supplemented with 10% FBS and transfected using Lipofectamine 3000 (Invitrogen, Carlsbad, CA) as per manufacturer’s instructions. Forty-eight hours post-transfection, cells with either the empty vector, mutant or WT Myc-tagged LRIT3 were harvested, proteins extracted using RIPA buffer with 1% protease and phosphatase inhibitor cocktail (Thermo Fisher Scientific, Waltham, MA), and run in a Western blot as detailed above. For immunocytochemistry, 48 hours post-transfected cells grown on poly D-lysine treated coverslips were fixed in 4% paraformaldehyde and permeabilised using 0.2% Triton X-100. The cells were then blocked for 15 min with 5% BSA and 2.5% fish gelatin prepared in PBS-T (PBS containing 0.1% Tween20). After washing, the cells were labelled with the appropriate antibodies for 1 hour following the manufacturer’s dilution instructions, washed and then labelled with an Alexa dye-tagged secondary antibody (Alexa Fluor Dyes, Molecular Probes, Invitrogen, Carlsbad, CA) at 1:200 dilution. The nuclei were stained with DAPI. The cells were mounted using Gelvatol mounting medium (Sigma Aldrich, St. Louis, MO), and imaged by confocal microscopy (Nikon, Instruments Inc., Melville, NY).

## Supplementary information

Supplementary Info

## Data Availability

The whole-genome sequencing datasets generated and analysed during the current study are available in the European Nucleotide Archive (links to datasets: https://www.ebi.ac.uk/ena/data/view/ERS3011607; https://www.ebi.ac.uk/ena/data/view/ERS3011608; https://www.ebi.ac.uk/ena/data/view/ERS3011609; https://www.ebi.ac.uk/ena/data/view/ERS3011610). All data generated or analysed during this study are included in this published article (and its Supplementary Information files).
