# Supplementary material for: Genome-wide association study and whole-genome sequencing identify a deletion in LRIT3 associated with canine congenital stationary night blindness
Source: Sci Rep. 2019 Oct 2;9:14166. doi: 10.1038/s41598-019-50573-7 (PMC6775105; doi:10.1038/s41598-019-50573-7)
Supplement: Supplementary file 1 — Supplementary Info [file 41598_2019_50573_MOESM1_ESM.pdf]

Genome-wide association study and whole-genome sequencing identify a deletion in *LRIT3* associated with canine congenital stationary night blindness

Rueben G. Das[1], Doreen Becker[1,2], Vidhya Jagannathan[3], Orly Goldstein[4], Evelyn Santana[1], Kendall Carlin[1], Raghavi Sudharsan[1], Tosso Leeb[3], Yuji Nishizawa[5], Mineo Kondo[6], Gustavo D. Aguirre[1], Keiko Miyadera[1*]

[1] Department of Clinical Sciences and Advanced Medicine, School of Veterinary Medicine, University of Pennsylvania, Pennsylvania, United States of America

[2] Institute of Genome Biology, Leibniz Institute for Farm Animal Biology, Dummerstorf, Germany

[3] Institute of Genetics, University of Bern, Bern, Switzerland

[4] Baker Institute for Animal Health, College of Veterinary Medicine, Cornell University, Ithaca, New York, United States of America

[5]Department of Biomedical Sciences, Chubu University, Kasugai, Aichi, Japan

[6]Department of Ophthalmology, Mie University Graduate School of Medicine, Tsu, Mie, Japan.

* Corresponding author

E-mail: kmiya@upenn.edu (K.M.)

	Parent-pup group 1				Parent-pup group 2								Parent-pup group 3						Parent-pup group 4						
	Dam	Pups		Sire	Dam	Pups						Sire	Dam	Pups				Sire	Dam	Pups					Sire
CFA32 position (CanFam3.1)	2F4	5F1	5F3	2M2	2F3	6F2*	6F4	6M1	6F1	6F3	6M2	3M2†	2F2	7F3	7F1	7M1	7M2	3M3	6F2*	9F1	9F2	9M1	9M2	9M3	3M2†
27,646,397	G G	G G	G G	G G	G G	G G	G G	G G	A G	A G	A G	A G	G G	G G	G G	G G	G G	G G	G G	G A	G G	A G	A G	A G	A G
27,708,968	A A	A A	A A	A A	A A	A A	A A	A A	G A	G A	G A	G A	A A	A A	A A	A A	A A	A A	A A	A G	A A	G A	G A	G A	G A
27,728,263	A A	A A	A A	A A	A A	A A	A A	A A	G A	G A	G A	G A	A A	A A	A A	A A	A A	A A	A A	A G	A A	G A	G A	G A	G A
27,741,326	G G	G G	G G	G G	G G	G G	G G	G G	A G	A G	A G	A G	G G	G G	G G	G G	G G	G G	G G	G A	G G	A G	A G	A G	A G
**27,752,177**	G G	G G	G G	G G	G G	G G	G G	G G	A G	A G	A G	A G	G G	G G	G G	G G	G G	G G	G G	G A	G G	A G	A G	A G	A G
27,794,707	G G	G G	G G	G G	G G	G G	G G	G G	G G	G G	G G	G G	G G	G G	A G	A G	A G	A G	G G	G G	G G	G G	G G	G G	G G
28,104,710	G G	G G	G G	G G	G G	G G	G G	G G	G G	G G	G G	G G	G G	G G	A G	A G	A G	A G	G G	G G	G G	G G	G G	G G	G G
28,118,831	G G	G G	G G	G G	G G	G G	G G	G G	A G	A G	A G	A G	G G	G G	G G	G G	G G	G G	G G	G G	G G	A G	A G	A G	A G
28,138,522	T T	T T	T T	T T	T T	T T	T T	T T	T T	T T	T T	T T	T T	T T	A T	A T	A T	A T	T T	T T	T T	T T	T T	T T	T T
28,165,875	A A	A A	A A	A A	A A	A A	A A	A A	A A	A A	A A	A A	A A	A A	G A	G A	G A	G A	A A	A A	A A	A A	A A	A A	A A
28,231,479	A A	A A	A A	A A	A A	A A	A A	A A	A A	A A	A A	A A	A A	A A	C A	C A	C A	C A	A A	A A	A A	A A	A A	A A	A A
28,250,646	A A	A A	A A	A A	A A	A A	A A	A A	A A	A A	A A	A A	A A	A A	G A	G A	G A	G A	A A	A A	A A	A A	A A	A A	A A
28,274,625	C C	C C	C C	C C	C C	C C	C C	C C	C C	C C	C C	C C	C C	C C	A C	A C	A C	A C	C C	C C	C C	C C	C C	C C	C C
28,357,578	A A	A A	A A	A A	A A	A A	A A	A A	G A	G A	G A	G A	A A	A A	G A	G A	G A	G A	A A	A A	A A	G A	G A	G A	G A
28,365,335	A A	A A	A A	A A	A A	A A	A A	A A	C A	C A	C A	C A	A A	A A	C A	C A	C A	C A	A A	A A	A A	C A	C A	C A	C A
28,375,105	C C	C C	C C	C C	C C	C C	C C	C C	G C	G C	G C	G C	C C	C C	G C	G C	G C	G C	C C	C C	C C	G C	G C	G C	G C
28,399,899	G G	G G	G G	G G	G G	G G	G G	G G	A G	A G	A G	A G	G G	G G	A G	A G	A G	A G	G G	G G	G G	A G	A G	A G	A G
28,432,837	A A	A A	A A	A A	A A	A A	A A	A A	A A	A A	A A	A A	A A	A A	G A	G A	G A	G A	A A	A A	A A	A A	A A	A A	A A
28,519,290	G G	G G	G G	G G	G G	G G	G G	G G	A G	A G	A G	A G	G G	G G	G G	G G	G G	G G	G G	G G	G G	A G	A G	A G	A G
28,539,559	G G	G G	G G	G G	G G	G G	G G	G G	A G	A G	A G	A G	G G	G G	A G	A G	A G	A G	G G	G G	G G	A G	A G	A G	A G
28,543,784	A A	A A	A A	A A	A A	A A	A A	A A	G A	G A	G A	G A	A A	A A	G A	G A	G A	G A	A A	A A	A A	G A	G A	G A	G A
28,577,006	G G	G G	G G	G G	G G	G G	G G	G G	A G	A G	A G	A G	G G	G G	G G	G G	G G	G G	G G	G G	G G	A G	A G	A G	A G
28,589,415	A A	A A	A A	A A	A A	A A	A A	A A	A A	A A	A A	A A	A A	A A	G A	G A	G A	G A	A A	A A	A A	A A	A A	A A	A A
28,647,658	G G	G G	G G	G G	G G	G G	G G	G G	G G	G G	G G	G G	G G	G G	A G	A G	A G	A G	G G	G G	G G	G G	G G	G G	G G
28,685,292	A A	A A	A A	A A	A A	A A	A A	A A	G A	G A	G A	G A	A A	A A	G A	G A	G A	G A	A A	A A	A A	G A	G A	G A	G A
28,702,140	A A	A A	A A	A A	A A	A A	A A	A A	A A	A A	A A	A A	A A	A A	G A	G A	G A	G A	A A	A A	A A	A A	A A	A A	A A
28,707,403	G G	G G	G G	G G	G G	G G	G G	G G	G G	G G	G G	G G	G G	G G	A G	A G	A G	A G	G G	G G	G G	G G	G G	G G	G G
28,717,263	A A	A A	A A	A A	A A	A A	A A	A A	A A	A A	A A	A A	A A	A A	G A	G A	G A	G A	A A	A A	A A	A A	A A	A A	A A
28,799,548	G G	G G	G G	G G	G G	G G	G G	G G	A G	A G	A G	A G	G G	G G	A G	A G	A G	A G	G G	G G	G G	A G	A G	A G	A G
28,811,735	A A	A A	A A	A A	A A	A A	A A	A A	G A	G A	G A	G A	A A	A A	A A	A A	A A	A A	A A	A A	A A	G A	G A	G A	G A
28,818,657	G G	G G	G G	G G	G G	G G	G G	G G	G G	G G	G G	G G	G G	G G	A G	A G	A G	A G	G G	G G	G G	G G	G G	G G	G G
28,840,988	G G	G G	G G	G G	G G	G G	G G	G G	G G	G G	G G	G G	G G	G G	A G	A G	A G	A G	G G	G G	G G	G G	G G	G G	G G
28,869,183	G G	G G	G G	G G	G G	G G	G G	G G	G G	G G	G G	G G	G G	G G	A G	A G	A G	A G	G G	G G	G G	G G	G G	G G	G G
28,882,948	G G	G G	G G	G G	G G	G G	G G	G G	G G	G G	G G	G G	G G	G G	A G	A G	A G	A G	G G	G G	G G	G G	G G	G G	G G
28,898,832	G G	G G	G G	G G	G G	G G	G G	G G	G G	G G	G G	G G	G G	G G	A G	A G	A G	A G	G G	G G	G G	G G	G G	G G	G G
28,900,743	G G	G G	G G	G G	G G	G G	G G	G G	G G	G G	G G	G G	G G	G G	A G	A G	A G	A G	G G	G G	G G	G G	G G	G G	G G
28,916,386	C C	C C	C C	C C	C C	C C	C C	C C	C C	C C	C C	C C	C C	C C	A C	A C	A C	A C	C C	C C	C C	C C	C C	C C	C C
28,933,783	A A	A A	A A	A A	A A	A A	A A	A A	G A	G A	G A	G A	A A	A A	G A	G A	G A	G A	A A	A A	A A	G A	G A	G A	G A
28,979,854	G G	G G	G G	G G	G G	G G	G G	G G	A G	A G	A G	A G	G G	G G	A G	A G	A G	A G	G G	G G	G G	A G	A G	A G	A G
28,999,310	G G	G G	G G	G G	G G	G G	G G	G G	G G	G G	G G	G G	G G	G G	A G	A G	A G	A G	G G	G G	G G	G G	G G	G G	G G
29,098,651	A A	A A	A A	A A	A A	A A	A A	A A	G A	G A	G A	G A	A A	A A	A A	A A	A A	A A	A A	A A	A A	G A	G A	G A	G A
29,141,932	G G	G G	G G	G G	G G	G G	G G	G G	A G	A G	A G	A G	G G	G G	G G	G G	G G	G G	G G	G G	G G	A G	A G	A G	A G
29,161,172	G G	G G	G G	G G	G G	G G	G G	G G	A G	A G	A G	A G	G G	G G	G G	G G	G G	G G	G G	G G	G G	A G	A G	A G	A G
29,215,206	G G	G G	G G	G G	G G	G G	G G	G G	G G	G G	G G	G G	G G	G G	A G	A G	A G	A G	G G	G G	G G	G G	G G	G G	G G
29,215,490	A A	A A	A A	A A	A A	A A	A A	A A	A A	A A	A A	A A	A A	A A	T A	T A	T A	T A	A A	A A	A A	A A	A A	A A	A A
29,349,819	A A	A A	A A	A A	A A	A A	A A	A A	G A	G A	G A	G A	A A	A A	A A	A A	A A	A A	A A	A A	A A	G A	G A	G A	G A
29,374,589	A A	A A	A A	A A	A A	A A	A A	A A	G A	G A	G A	G A	A A	A A	A A	A A	A A	A A	A A	A A	A A	G A	G A	G A	G A
29,419,417	G G	G G	G G	G G	G G	G G	G G	G G	A G	A G	A G	A G	G G	G G	G G	G G	G G	G G	G G	G G	G G	A G	A G	A G	A G
29,474,606	G G	G G	G G	G G	G G	G G	G G	G G	A G	A G	A G	A G	G G	G G	G G	G G	G G	G G	G G	G G	G G	A G	A G	A G	A G
29,488,133	G G	G G	G G	G G	G G	G G	G G	G G	A G	A G	A G	A G	G G	G G	G G	G G	G G	G G	G G	G G	G G	A G	A G	A G	A G
29,523,416	G G	G G	G G	G G	G G	G G	G G	G G	A G	A G	A G	A G	G G	G G	G G	G G	G G	G G	G G	G G	G G	A G	A G	A G	A G
29,545,160	A A	A A	A A	A A	A A	A A	A A	A A	C A	C A	C A	C A	A A	A A	A A	A A	A A	A A	A A	A A	A A	C A	C A	C A	C A
29,556,711	G G	G G	G G	G G	G G	G G	G G	G G	A G	A G	A G	A G	G G	G G	G G	G G	G G	G G	G G	G G	G G	A G	A G	A G	A G
29,627,092	A A	A A	A A	A A	A A	A A	A A	A A	A A	A A	A A	A A	A A	A A	G A	G A	G A	G A	A A	A A	A A	A A	A A	A A	A A
29,643,731	C C	C C	C C	C C	C C	C C	C C	C C	C C	C C	C C	C C	C C	C C	A C	A C	A C	A C	C C	C C	C C	C C	C C	C C	C C
29,668,146	A A	A A	A A	A A	A A	A A	A A	A A	A A	A A	A A	A A	A A	A A	G A	G A	G A	G A	A A	A A	A A	A A	A A	A A	A A
29,689,318	A A	A A	A A	A A	A A	A A	A A	A A	A A	A A	A A	A A	A A	A A	G A	G A	G A	G A	A A	A A	A A	A A	A A	A A	A A
29,694,240	A A	A A	A A	A A	A A	A A	A A	A A	A A	A A	A A	A A	A A	A A	G A	G A	G A	G A	A A	A A	A A	A A	A A	A A	A A
29,705,999	G G	G G	G G	G G	G G	G G	G G	G G	G G	G G	G G	G G	G G	G G	A G	A G	A G	A G	G G	G G	G G	G G	G G	G G	G G
29,762,550	G G	G G	G G	G G	G G	G G	G G	G G	G G	G G	G G	G G	G G	G G	A G	A G	A G	A G	G G	G G	G G	G G	G G	G G	G G
29,799,313	G G	G G	G G	G G	G G	G G	G G	G G	G G	G G	G G	G G	G G	G G	A G	A G	A G	A G	G G	G G	G G	G G	G G	G G	G G
29,848,255	C C	C C	C C	C C	C C	C C	C C	C C	C C	C C	C C	C C	C C	C C	A C	A C	A C	A C	C C	C C	C C	C C	C C	C C	C C
29,855,210	A A	A A	A A	A A	A A	A A	A A	A A	A A	A A	A A	A A	A A	A A	G A	G A	G A	G A	A A	A A	A A	A A	A A	A A	A A
29,883,318	G G	G G	G G	G G	G G	G G	G G	G G	G G	G G	G G	G G	G G	G G	A G	A G	A G	A G	G G	G G	G G	G G	G G	G G	G G
29,891,340	A A	A A	A A	A A	A A	A A	A A	A A	A A	A A	A A	A A	A A	A A	G A	G A	G A	G A	A A	A A	A A	A A	A A	A A	A A
29,892,939	G G	G G	G G	G G	G G	G G	G G	G G	G G	G G	G G	G G	G G	G G	A G	A G	A G	A G	G G	G G	G G	G G	G G	G G	G G
29,904,874	G G	G G	G G	G G	G G	G G	G G	G G	G G	G G	G G	G G	G G	G G	A G	A G	A G	A G	G G	G G	G G	G G	G G	G G	G G
29,915,018	G G	G G	G G	G G	G G	G G	G G	G G	G G	G G	G G	G G	G G	G G	A G	A G	A G	A G	G G	G G	G G	G G	G G	G G	G G
29,963,161	G G	G G	G G	G G	G G	G G	G G	G G	G G	G G	G G	G G	G G	G G	A G	A G	A G	A G	G G	G G	G G	G G	G G	G G	G G
29,983,332	A A	A A	A A	A A	A A	A A	A A	A A	A A	A A	A A	A A	A A	A A	G A	G A	G A	G A	A A	A A	A A	A A	A A	A A	A A
30,064,792	A A	A A	A A	A A	A A	A A	A A	A A	A A	A A	A A	A A	A A	A A	C A	C A	C A	C A	A A	A A	A A	A A	A A	A A	A A
30,097,173	A A	A A	A A	A A	A A	A A	A A	A A	A A	A A	A A	A A	A A	A A	G A	G A	G A	G A	A A	A A	A A	A A	A A	A A	A A
30,126,952	A A	A A	A A	A A	A A	A A	A A	A A	A A	A A	A A	A A	A A	A A	G A	G A	G A	G A	A A	A A	A A	A A	A A	A A	A A
30,132,313	G G	G G	G G	G G	G G	G G	G G	G G	G G	G G	G G	G G	G G	G G	A G	A G	A G	A G	G G	G G	G G	G G	G G	G G	G G
30,247,125	C C	C C	C C	C C	C C	C C	C C	C C	C C	C C	C C	C C	C C	C C	A C	A C	A C	A C	C C	C C	C C	C C	C C	C C	C C
30,263,648	A A	A A	A A	A A	A A	A A	A A	A A	A A	A A	A A	A A	A A	A A	G A	G A	G A	G A	A A	A A	A A	A A	A A	A A	A A
30,309,957	G G	G G	G G	G G	G G	G G	G G	G G	G G	G G	G G	G G	G G	G G	A G	A G	A G	A G	G G	G G	G G	G G	G G	G G	G G
30,521,507	G G	G G	G G	G G	G G	G G	G G	G G	G G	G G	G G	G G	G G	G G	A G	A G	A G	A G	G G	G G	G G	G G	G G	G G	G G
30,524,319	A A	A A	A A	A A	A A	A A	A A	A A	A A	A A	A A	A A	A A	A A	G A	G A	G A	G A	A A	A A	A A	A A	A A	A A	A A
30,536,559	G G	G G	G G	G G	G G	G G	G G	G G	G G	G G	G G	G G	G G	G G	A G	A G	A G	A G	G G	G G	G G	G G	G G	G G	G G
30,572,696	A A	A A	A A	A A	A A	A A	A A	A A	A A	A A	A A	A A	A A	A A	G A	G A	G A	G A	A A	A A	A A	A A	A A	A A	A A
30,586,942	C C	C C	C C	C C	C C	C C	C C	C C	C C	C C	C C	C C	C C	C C	A C	A C	A C	A C	C C	C C	C C	C C	C C	C C	C C
30,594,912	G G	G G	G G	G G	G G	G G	G G	G G	G G	G G	G G	G G	G G	G G	A G	A G	A G	A G	G G	G G	G G	G G	G G	G G	G G
30,603,746	A A	A A	A A	A A	A A	A A	A A	A A	A A	A A	A A	A A	A A	A A	G A	G A	G A	G A	A A	A A	A A	A A	A A	A A	A A
30,718,156	G G	G G	G G	G G	G G	G G	G G	G G	G G	G G	G G	G G	G G	G G	A G	A G	A G	A G	G G	G G	G G	G G	G G	G G	G G
30,799,197	A A	A A	A A	A A	A A	A A	A A	A A	A A	A A	A A	A A	A A	A A	G A	G A	G A	G A	A A	A A	A A	A A	A A	A A	A A
30,810,763	A A	A A	A A	A A	A A	A A	A A	A A	A A	A A	A A	A A	A A	A A	C A	C A	C A	C A	A A	A A	A A	A A	A A	A A	A A
30,936,090	A A	A A	A A	A A	A A	A A	A A	A A	A A	A A	A A	A A	A A	A A	G A	G A	G A	G A	A A	A A	A A	A A	A A	A A	A A
30,980,961	A A	A A	A A	A A	A A	A A	A A	A A	A A	A A	A A	A A	A A	A A	G A	G A	G A	G A	A A	A A	A A	A A	A A	A A	A A
31,148,597	C C	C C	C C	C C	C C	C C	C C	C C	C C	C C	C C	C C	C C	C C	G C	G C	G C	G C	C C	C C	C C	C C	C C	C C	C C
31,169,274	A A	A A	A A	A A	A A	A A	A A	A A	A A	A A	A A	A A	A A	A A	G A	G A	G A	G A	A A	A A	A A	A A	A A	A A	A A
31,206,079	A A	A A	A A	A A	A A	A A	A A	A A	A A	A A	A A	A A	A A	A A	C A	C A	C A	C A	A A	A A	A A	A A	A A	A A	A A
31,295,694	G G	G G	G G	G G	G G	G G	G G	G G	G G	G G	G G	G G	G G	G G	C G	C G	C G	C G	G G	G G	G G	G G	G G	G G	G G
31,311,408	C C	C C	C C	C C	C C	C C	C C	C C	C C	C C	C C	C C	C C	C C	A C	A C	A C	A C	C C	C C	C C	C C	C C	C C	C C
31,318,484	A A	A A	A A	A A	A A	A A	A A	A A	A A	A A	A A	A A	A A	A A	G A	G A	G A	G A	A A	A A	A A	A A	A A	A A	A A
31,471,171	G G	G G	G G	G G	G G	G G	G G	G G	G G	G G	G G	G G	G G	G G	A G	A G	A G	A G	G G	G G	G G	G G	G G	G G	G G
31,510,703	G G	G G	G G	G G	G G	G G	G G	G G	G G	G G	G G	G G	G G	G G	C G	C G	C G	C G	G G	G G	G G	G G	G G	G G	G G
31,522,041	A A	A A	A A	A A	A A	A A	A A	A A	A A	A A	A A	A A	A A	A A	G A	G A	G A	G A	A A	A A	A A	A A	A A	A A	A A
31,545,851	A A	A A	A A	A A	A A	A A	A A	A A	A A	A A	A A	A A	A A	A A	G A	G A	G A	G A	A A	A A	A A	A A	A A	A A	A A
31,603,017	A A	A A	A A	A A	A A	A A	A A	A A	A A	A A	A A	A A	A A	A A	G A	G A	G A	G A	A A	A A	A A	A A	A A	A A	A A
31,655,411	A A	A A	A A	A A	A A	A A	A A	A A	A A	A A	A A	A A	A A	A A	C A	C A	C A	C A	A A	A A	A A	A A	A A	A A	A A
31,685,237	T T	T T	T T	T T	T T	T T	T T	T T	T T	T T	T T	T T	T T	T T	A T	A T	A T	A T	T T	T T	T T	T T	T T	T T	T T
31,742,568	A A	A A	A A	A A	A A	A A	A A	A A	A A	A A	A A	A A	A A	A A	G A	G A	G A	G A	A A	A A	A A	A A	A A	A A	A A
31,765,254	G G	G G	G G	G G	G G	G G	G G	G G	G G	G G	G G	G G	G G	G G	A G	A G	A G	A G	G G	G G	G G	G G	G G	G G	G G
31,785,146	A A	A A	A A	A A	A A	A A	A A	A A	A A	A A	A A	A A	A A	A A	C A	C A	C A	C A	A A	A A	A A	A A	A A	A A	A A
31,822,312	A A	A A	A A	A A	A A	A A	A A	A A	A A	A A	A A	A A	A A	A A	G A	G A	G A	G A	A A	A A	A A	A A	A A	A A	A A
31,996,425	G G	G G	G G	G G	G G	G G	G G	G G	G G	G G	G G	G G	G G	G G	A G	A G	A G	A G	G G	G G	G G	G G	G G	G G	G G
32,010,665	G G	G G	G G	G G	G G	G G	G G	G G	G G	G G	G G	G G	G G	G G	A G	A G	A G	A G	G G	G G	G G	G G	G G	G G	G G
32,204,043	A A	A A	A A	A A	A A	A A	A A	A A	C A	C A	C A	C A	A A	A A	C A	C A	C A	C A	A A	A A	A A	C A	C A	C A	C A
32,217,460	A A	A A	A A	A A	A A	A A	A A	A A	G A	G A	G A	G A	A A	A A	G A	G A	G A	G A	A A	A A	A A	G A	G A	G A	G A
32,226,098	G G	G G	G G	G G	G G	G G	G G	G G	A G	A G	A G	A G	G G	G G	A G	A G	A G	A G	G G	G G	G G	A G	A G	A G	A G
32,235,122	G G	G G	G G	G G	G G	G G	G G	G G	A G	A G	A G	A G	G G	G G	A G	A G	A G	A G	G G	G G	G G	A G	A G	A G	A G
32,262,774	A A	A A	A A	A A	A A	A A	A A	A A	G A	G A	G A	G A	A A	A A	G A	G A	G A	G A	A A	A A	A A	G A	G A	G A	G A
**32,306,733**	A G	A G	G G	A G	A G	A A	A A	A A	G A	G A	G G	G A	A G	A G	G A	G A	G G	G A	A A	A G	A A	G A	G -	G -	G A
32,437,068	G A	G A	A A	G A	G A	G G	G G	G G	G G	G G	G A	G G	G A	G A	G G	G G	G A	G G	G G	G A	G G	G G	G G	G G	G G
32,463,865	A G	A G	G G	A G	A G	A A	A A	A A	A A	A A	A G	A A	A G	A G	A A	A A	A G	A A	A A	A G	A A	A A	A A	A A	A A

Supplementary Figure 1

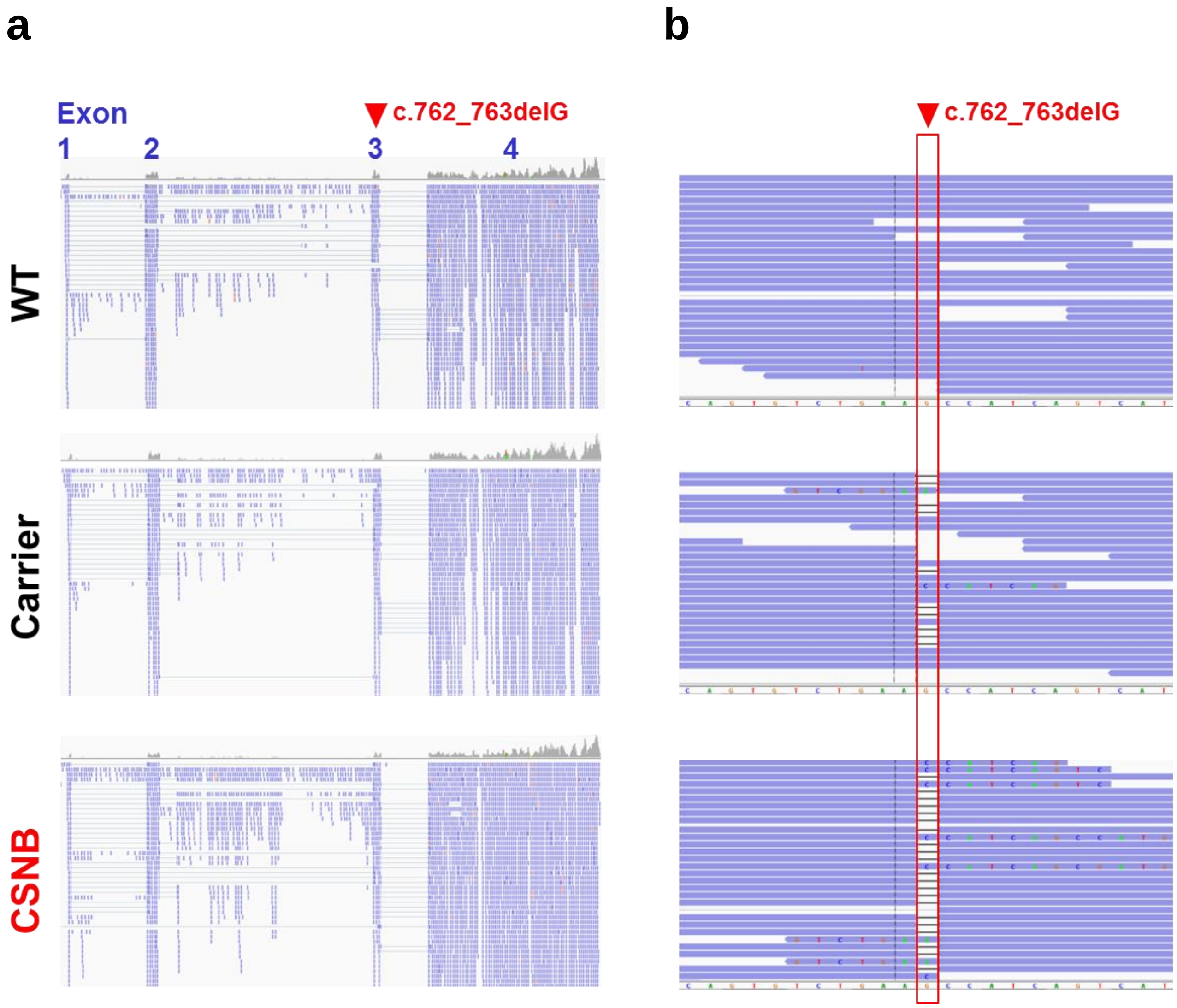

Supplementary Figure 2

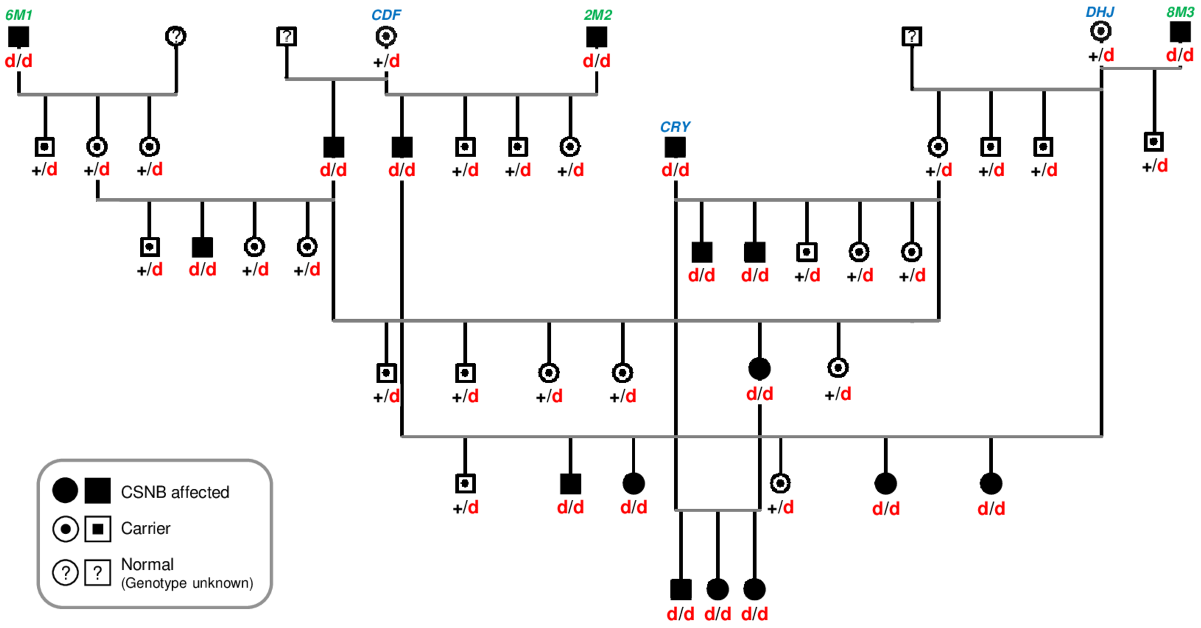

Supplementary Figure 3

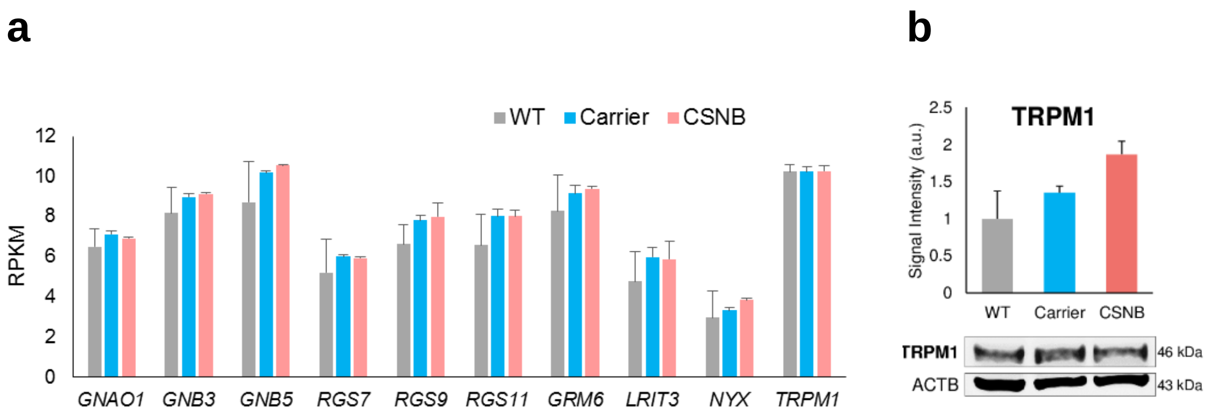

Supplementary Figure 4

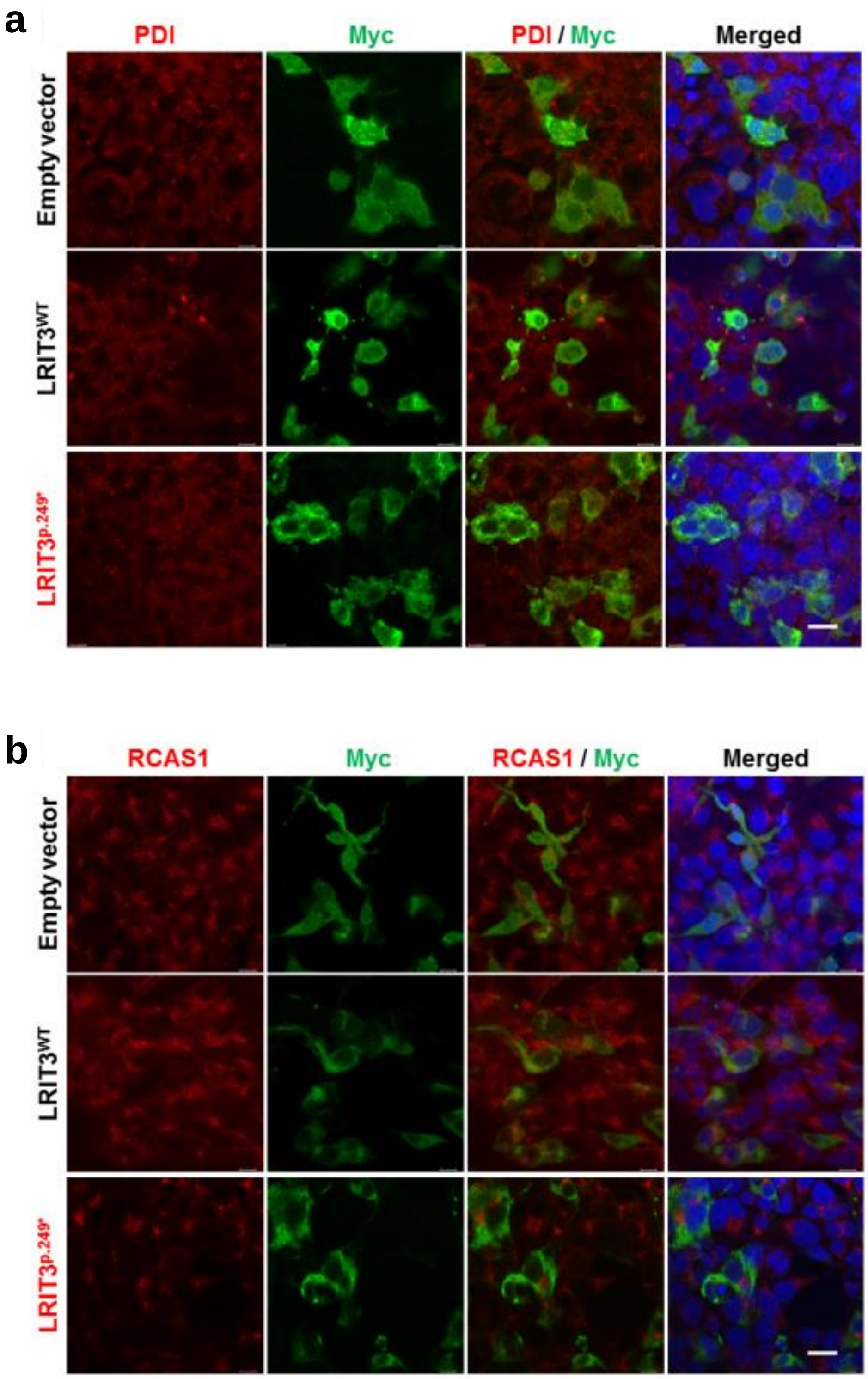

Supplementary Figure 5

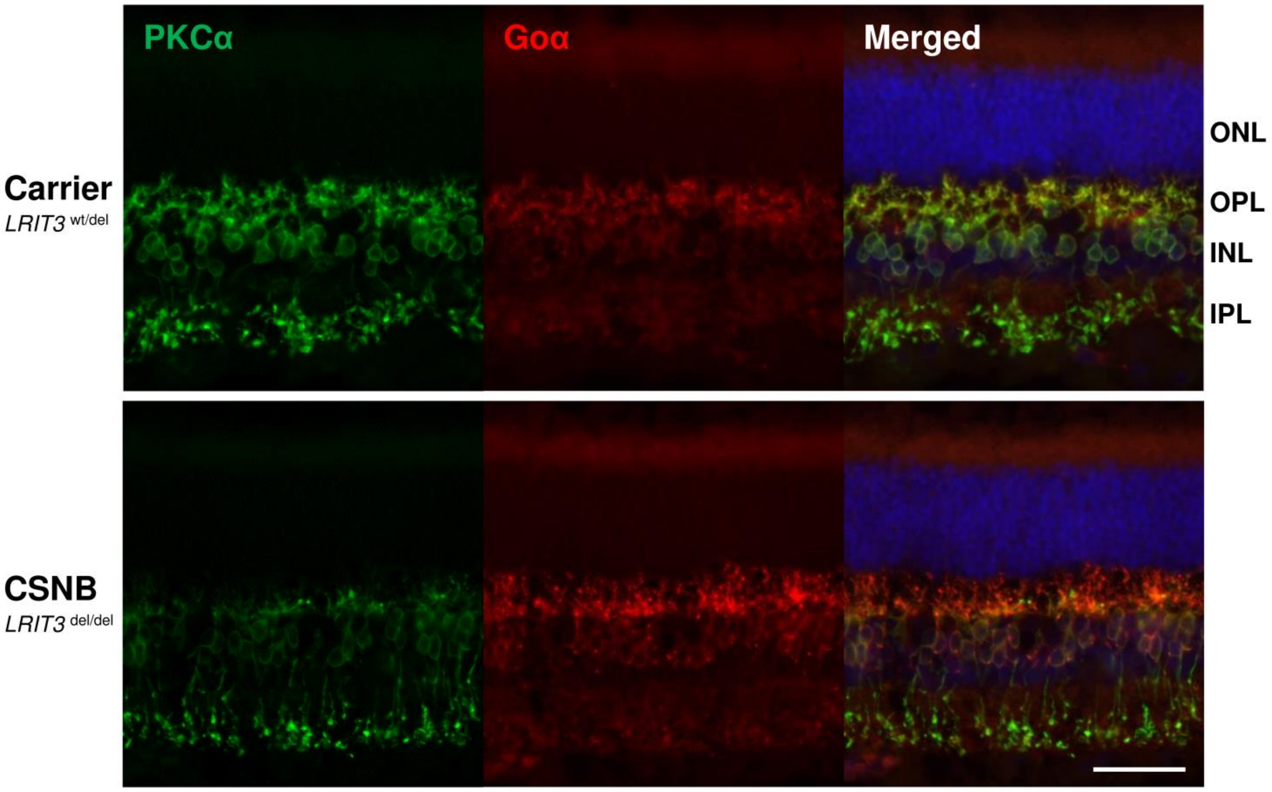

Supplementary Figure 6

a

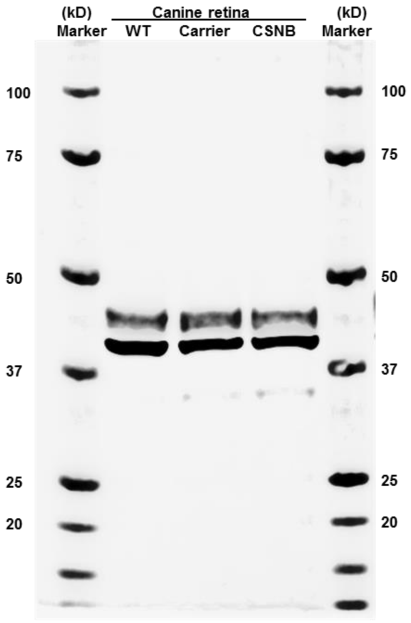

b

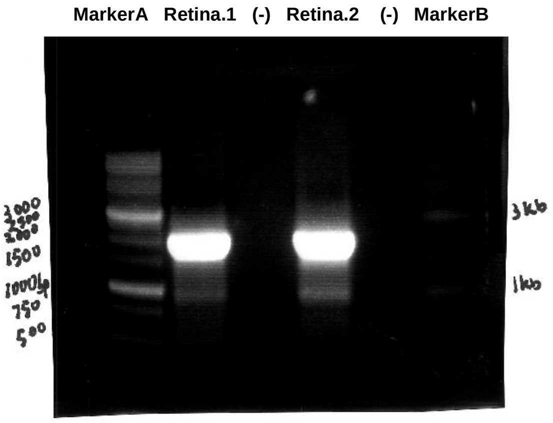

c

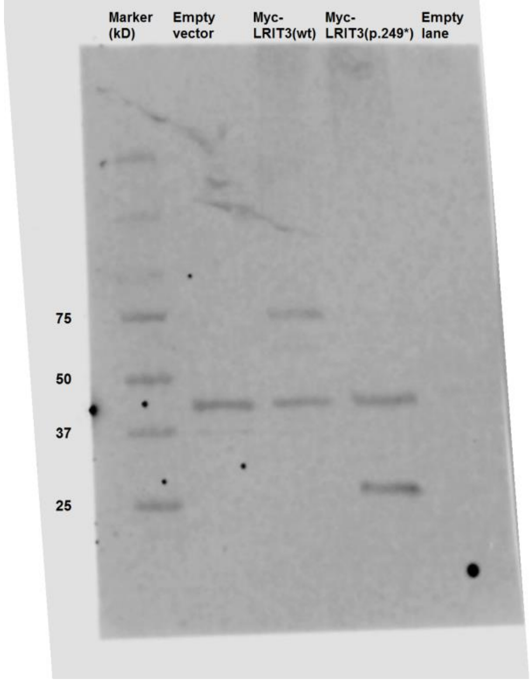

Supplementary Figure 7

Supplementary Information 1

a. Full-length isoform

1TAACCTTCAGAAGGAGCGATGCATCTCTCAGCATGTCTGTGCATTGTGCTTGTTAGCTTTTTGGAAAGAGGGAGCTGTTCAT
GTCCTTCACAGTGTACCTGCGATTATCATGGCAGAAATGACGGAACGGGATCAAG**2**GTCGGTGCTATGTAATGACCTGGATAT
GAATGAGGTCCCTATGAACTTCCCCGTGGACACTGTGAAGCTACGTATAGAGAAGACAGTCATCCGCAGAATCCCCGCAGAGG
CCTTCTACTACCTGGTGGAGCTCCAATACCTCTGGGTGACTTACAATTCCGTGGCCAGCCTTGATGCCAGCAGCTTTTACAAC
CTAAAGCAGCTGCATGAGTTGCGCTTGGATGGAAATTCTCTGGTTGCTTTCCCTTGGGCATCTCTGTTGGACATGCCCCATCT
GAGGACCCTGGATTTGCACAACAACAGAATAACCAGTGTGCCAAATGAGGTGGTCAGGTACCTGAAGAACCTTGCCTACTTGG
ATTTATCAAGCAACAGGCTAACCACACTACCACCAGATTTCCTGGAGAGCTGGTCCCACTTACTTACAACATCATTACGAAGC
CTGGACCTTACACCACGAAGAATTATTCTTG**3**GTCTGCAGGACAACCCTTGGTTCTGTGACTGTCACATTTCCAAAATGATCG
AGTTGTCAAAAGTTGCTGACCCTTCTATAGTGCTTCTTGATCCACTGATGATTTGTAGCGAACCTGAGCGCCTGACAGGGATT
TTGTTTCAGCGGGCTGAGTTGGAGCAGTGTCTGAAGCCATCAGTCATGACCTCGGCCACCCAGATCACATCTGCTCTGGGTAG
TAACGTTCTGCTGCGGTGTGATGCTACTGGCTACCCTACCCCACAGCTCACGTGGACCAGATCTGACAGCTCACCAGTTAATT
ATACAG**4**TAATTCAGGAATCTCCAGGGGAAGGAGTCAGATGGTCCATAATAAGCTTGACTGGTATTTCTTACAAGGATGCTGG
GGATTACAAATGTAAGGCCAAAAATTTGGCTGGAATGTCAGAAGCTATGGTTACTGTGACAGTGGTTGGTGTTGTCACAACCA
CCATATCATCAGAGACCTCTGAAAGAACTGGAGATCACCCTGAGGAAGAAGTCCAGCCAAGATCCAGATCTACATCCCCGCCT
GGTTCATTGTCATCTTCTTGGCCTTCCTCCTCCCCTGCTTCTTCCATTTTTCTTTCTAATTCTACTTCATCTCCTCCCCCCTC
TGCTTTCTTCTCCTTATCTCCTTTCCTCTCCTCTATTGTTTCTTCAACCACAGCTAGAAACACTGGAATTTCAACAAGCCCCA
CCATGGCCAGCCAGCAGTCACTCCAGCTCCACCCAGATGGGAAAAGAAATTTAAAGGTGGAGTTGGGTGGAAGTCAGCTTCCT
TCAGGTAGTGCAAGTAGAAAAGAAGAGTTGGCATTGTTGGATGAAGCAACGCCAGTGGAAACAAATACCACAATAGAGAATCT
CAGGGTAGTTGGTGAGACTGAAGAGAGTGTCACATTGATGTGGAACACTGTCAACACCACACAAAAGTCTGAGATGACAGTGT
TGTACTCCAAGTATGGTGAAAAGGACCTGCTGCTGCTGAATGCTGACTCCAGCAAGAACCAAGTAACCATAGATGGCTTGGAG
CCTGGTAGGCAATATATAGCATGTGTCTGTCCAAAAGGAATACCTCCCCAGAAAGACCAATGTATTACCTTTTCTACTGACAG
AGTTGAAAAGGAAGGTGATTCTCAAGTGTCTTTCCTTGTTGTGGTGAGCGGTACCGCCTGTGTTGTTGTCTTGCCATTGATAT
TTTTCCTGTTGTACAAAGTTTGCAAACTGCAATGTAGGTCAGAATCTTTCTGGGAAGATGACTTGGCAAAAGAAACTTATATC
CAATTTGAAACCCTGTCCCCCAGGTCTCAAAGTGTAGGGGAGCTTTGGACGCGAAGGCACAGCGATGATTCAGAAAAATTGCT
GCTTTGTTCTAGGTCAAGTGTAGAGTCTCAGATGACTTTTAAAAATGAAGGTTTGAGATCAGAGTATTATTGCTAAGATTTTA
CACATGAAC

b. Shorter isoform with exon 2 skipping

1TAACCTTCAGAAGGAGCGATGCATCTCTCAGCATGTCTGTGCATTGTGCTTGTTAGCTTTTTGGAAAGAGGGAGCTGTTCAT
GTCCTTCACAGTGTACCTGCGATTATCATGGCAGAAATGACGGAACGGGATCAAG**3**GTCTGCAGGACAACCCTTGGTTCTGTG
ACTGTCACATTTCCAAAATGATCGAGTTGTCAAAAGTTGCTGACCCTTCTATAGTGCTTCTTGATCCACTGATGATTTGTAGC
GAACCTGAGCGCCTGACAGGGATTTTGTTTCAGCGGGCTGAGTTGGAGCAGTGTCTGAAGCCATCAGTCATGACCTCGGCCAC
CCAGATCACATCTGCTCTGGGTAGTAACGTTCTGCTGCGGTGTGATGCTACTGGCTACCCTACCCCACAGCTCACGTGGACCA
GATCTGACAGCTCACCAGTTAATTATACAG**4**TAATTCAGGAATCTCCAGGGGAAGGAGTCAGATGGTCCATAATAAGCTTGAC
TGGTATTTCTTACAAGGATGCTGGGGATTACAAATGTAAGGCCAAAAATTTGGCTGGAATGTCAGAAGCTATGGTTACTGTGA
CAGTGGTTGGTGTTGTCACAACCACCATATCATCAGAGACCTCTGAAAGAACTGGAGATCACCCTGAGGAAGAAGTCCAGCCA
AGATCCAGATCTACATCCCCGCCTGGTTCATTGTCATCTTCTTGGCCTTCCTCCTCCCCTGCTTCTTCCATTTTTCTTTCTAA
TTCTACTTCATCTCCTCCCCCCTCTGCTTTCTTCTCCTTATCTCCTTTCCTCTCCTCTATTGTTTCTTCAACCACAGCTAGAA
ACACTGGAATTTCAACAAGCCCCACCATGGCCAGCCAGCAGTCACTCCAGCTCCACCCAGATGGGAAAAGAAATTTAAAGGTG
GAGTTGGGTGGAAGTCAGCTTCCTTCAGGTAGTGCAAGTAGAAAAGAAGAGTTGGCATTGTTGGATGAAGCAACGCCAGTGGA
AACAAATACCACAATAGAGAATCTCAGGGTAGTTGGTGAGACTGAAGAGAGTGTCACATTGATGTGGAACACTGTCAACACCA
CACAAAAGTCTGAGATGACAGTGTTGTACTCCAAGTATGGTGAAAAGGACCTGCTGCTGCTGAATGCTGACTCCAGCAAGAAC
CAAGTAACCATAGATGGCTTGGAGCCTGGTAGGCAATATATAGCATGTGTCTGTCCAAAAGGAATACCTCCCCAGAAAGACCA
ATGTATTACCTTTTCTACTGACAGAGTTGAAAAGGAAGGTGATTCTCAAGTGTCTTTCCTTGTTGTGGTGAGCGGTACCGCCT
GTGTTGTTGTCTTGCCATTGATATTTTTCCTGTTGTACAAAGTTTGCAAACTGCAATGTAGGTCAGAATCTTTCTGGGAAGAT
GACTTGGCAAAAGAAACTTATATCCAATTTGAAACCCTGTCCCCCAGGTCTCAAAGTGTAGGGGAGCTTTGGACGCGAAGGCA
CAGCGATGATTCAGAAAAATTGCTGCTTTGTTCTAGGTCAAGTGTAGAGTCTCAGATGACTTTTAAAAATGAAGGTTTGAGAT
CAGAGTATTATTGCTAAGATTTTACACATGAAC

Supplementary Table 1. Dogs from the Dog Biomedical Variant Database Consortium used for cross-referencing of whole-genome sequencing data.

**Breed**	**Number of animals**
Affenpinscher	1
Airdale Terrier	1
Alaskan Husky	2
Alaskan Malamute	1
Alpine Dachsbracke	1
American Bulldog	1
American Staffordshire Terrier	1
Australian Cattle Dog	2
Australian Terrier	1
Basset Hound	2
Beagle	1
Bearded Collie	11
Berger Blanc Suisse	1
Border Collie	27
Boxer	1
Bullmastiff	1
Bullterrier	1
Cairn Terrier	1
Cane Corso	1
Cavalier King Charles Spaniel	1
Central Asian Shepherd dog	1
Chihuahua	1
Chinese Indigenous Dog	28
Cocker Spaniel	1
Curly Coated Retriever	1
Dachshund	1
Dalmatian	3
Dandie Dinmont Terrier	1
Doberman Pinscher	3
Dogue de Bordeaux	3
Elo	1
Entlebucher Mountain Dog	8
Eurasier	2
Finnish Lapphund	1
French Bulldog	3
German Shepherd	14
German Wirehaired	1
Golden Retriever	4
Great Dane	1
Greater Swiss Mountain Dog	2
Heideterrier	1

Hovawart	1
Irish Soft Coated Wheaten Terrier	1
Irish Terrier	1
Italian Greyhound	1
Karelian Bear dog	1
Kromfohrländer	1
Kunming Dog	10
Labrador Retriever	3
Lagotto Romagnola	5
Landseer	2
Leonberger	41
Malinois	4
Miniature Bullterrier	1
Miniature Poodle	4
Miniature Schnauzer	10
Mixed breed	1
Norwich Terrier	4
Nova Scotia Duck Tolling Retriever	1
Old English Sheepdog	1
Perro de Agua Espa–ol	1
Pomeranian	1
Standard Poodle	1
Rhodesian Ridgeback	4
Rottweiler	2
Saluki	1
Siberian Husky	1
Sloughi	3
Swedish Vallhund	2
Tibetan Mastiff	10
Weimaraner	1
Welsh Springer Spaniel	2
West Highland White Terrier	6
Whippet	1
White Shepherd	1
Yorkshire Terrier	1
**Total**	**271**

Supplementary Table 2. Primary antibodies used in the study.

Marker	Host	Dilution (ICC)	Dilution (IHC)	Dilution (Western)	Source
Myc1	Mouse	1:200	-	1:1000	Clone 9E10 batch 20 (UPENN Cell Center)
ACTB	Mouse	-	-	1:10,000	Millipore MAB1501
	Rabbit	-	-	1:10,000	Abcam ab8227
PDI	Rabbit	1:200	-	-	3501S, Cell Signaling Technology
RCAS1	Rabbit	1:200	-	-	12290S, Cell Signaling Technology
LRIT3	Rabbit	-	1:100	Did not give specific band	Sigma HPA013454
Goα	Mouse	-	1:5,000	-	Millipore MAB 3073
PNA lectin (red/green)	NA	-	1:20	-	Molecular Probes L-32458 (red)/ L21409 (green)
TRPM1	Rabbit	-	-	1:1,000	Sigma, HPA014785

Legends to Supporting information

Supplementary Figure 1. Haplotypes across the CSNB critical interval on canine chromosome 32 (CFA32). Haplotypes based on informative SNVs identified from the Illumina chip in all the dogs typed. The population consist of four parent-pup groups. The IDs of CSNB affected dogs are highlighted in orange. The SNVs highlighted in yellow represent alternative alleles. The SNV positions representing the disease interval based on the extension of the homozygosity block are highlighted in red. *†These animals appear twice in the figure.

Supplementary Figure 2. Position of the CSNB genetic variant in *LRIT3* shown in RNA-seq. RNA-seq reads from WT, carrier, and CSNB canine retinas aligned to the CanFam3.1 reference genome and viewed using the IGV software. (**a**) The region displayed encompasses the entire *LRIT3* that consists of 4 exons. The CSNB-associated deletion resides in exon 3 (red triangle). The gross exon structure indicated by sequence reads and their depths are comparable across the three groups demonstrating that the mutant *LRIT3* transcript escapes nonsense-mediated decay. (**b**) A closer view of the deletion site in *LRIT3* demonstrates the presence of the 1bp deletion in the carrier and CSNB at heterozygous and homozygous states, respectively. Note that where IGV considers that there might be a mismatch with the reference sequence, it displays the read with a colour-coded nucleotide. As a result, in the CSNB retina, some of the reads appear to override the 1bp deletion site as a mismatched base, where the other correctly aligned reads are replaced by a black bar. The colour-coded read overriding the 1bp deletion site is flanked on one side with apparent mismatches. Close inspection of these sequences shows that these reads indeed harbour the 1bp deletion. Two such misalignments are also found in the carrier.

Supplementary Figure 3. Segregation of CSNB in a satellite canine research colony and association with the *LRIT3* variant. The pedigree of the newly developed sattellite canine research colony segregating CSNB is shown. The colony was founded by animals (ID shown in green) from the original colony [37] . Additional founders included Beagle dogs (ID shown in blue) obtained from a laboratory animal vendor and that were identified as carriers or affected for CSNB. The alleles '+'

and 'd' represents wild type and the deletion variant of *LRIT3*, respectively. The *LRIT3* variant segregated completely with the recessive CSNB phenotype.

Supplementary Figure 4. RNA-seq analysis of genes involved in the mGluR6 pathway and Western analysis of selected proteins. (**a**) RNA-seq analysis showing lack of differential expression of the mGluR6 pathway genes between WT, carrier, and CSNB canine retinas (n=3 each). Error bars represent standard deviation. (**b**) Comparable expression of TRPM1 by Western blot. Canine retinal protein extracts from WT (n=3), obligate carrier (n=3), and CSNB affected (n=3) animals were used for Western blot. Quantitation of TRPM1, an ON-BC protein involved in the mGluR6 pathway, showed no significant change in expression among the different phenotypic groups. Error bars represent standard deviation. Representative western blot images are shown below the corresponding graph. ACTB was used as the loading control. The molecular weight (kDa) of specific immunolabelled bands are indicated on the side. The images of bands for the target protein and ACTB are taken from the same blot and each image has been cropped as delineated by black dividing lines. Any adjustments for image intensity for optimal visualization were applied uniformly across the blot.

Supplementary Figure 5. Comparable localization of WT and mutant LRIT3 *in vitro*. (**a**, **b**) Immunocytochemistry of COS1 cells overexpressing Myc-tagged WT or mutant (p.249*) LRIT3 indicates non-retention of either product in the endoplasmic reticulum (**a**; PDI) or Golgi (**b**; RCAS1) using cell organelle specific markers. These preliminary data propose a potentially comparable subcellular expression pattern of WT and truncated LRIT3. Nuclei were stained with DAPI. Scale bar, 20μm.

Supplementary Figure 6. Altered PKCα labelling in the canine CSNB retina. Immunolabelling with rod ON-BC marker PKCα shows reduced signal in the somata and the dendrites of the CSNB retina in comparison to the carrier retina. Scale bar, 40μm.

Supplementary Figure 7. Complete images of the gel and blot presented in Supplementary Figure 4 and Figure 4. (**a**) The original blot image which has been cropped in Supplementary Figure 4b is shown. (**b**) The entire available RT-PCR gel image presented in Figure 4a is shown. While the

edges of the gel are not delineated in this photograph, all positive bands appearing on the gel have been captured in the image. Note that the second ('Retina.1') and fourth ('Retina.2') lanes are products of identical RT-PCR reactions carried out in two separate tubes, while the third and fifth lanes represent no-template PCR controls. (**c**) The full-length blot image which has been cropped in Figure 4b is shown.

Supplementary Information 1. Experimentally confirmed sequences of canine *LRIT3* cDNA transcripts. The sequences for the predominant, full-length transcript (**a**) and a minor and shorter isoform with exon 2 skipping (**b**) are derived from and cross-checked between RT-PCR and RNA-seq data obtained from canine retina. Exon numbers are shown in blue. Start and stop codons are outlined. Note that the shorter isoform is in-frame by utilizing an alternative start codon, and harbours the disease variant as well. The position of the c.762_763delG disease variant is highlighted in red.
